# CircHipk3 serves a dual role in macrophage pyroptosis by promoting NLRP3 transcription and inhibition of autophagy to induce abdominal aortic aneurysm formation

**DOI:** 10.1002/ctm2.70102

**Published:** 2024-11-27

**Authors:** Donghua Cai, Chuling Li, Yingyuan Zhang, Sisi He, Yihai Guo, Wangjun Liao, Yulin Liao, Jianping Bin, Xiang He

**Affiliations:** ^1^ Department of Cardiology, State Key Laboratory of Organ Failure Research, Nanfang Hospital Southern Medical University Guangzhou China; ^2^ Guangdong Provincial Key Laboratory of Cardiac Function and Microcirculation Guangzhou China; ^3^ Department of Cardiology, Guangzhou First People's Hospital, School of Medicine South China University of Technology Guangzhou China; ^4^ Department of Oncology, Nanfang Hospital Southern Medical University Guangzhou China

**Keywords:** abdominal aortic aneurysm, circHipk3, macrophage, NLRP3, pyroptosis

## Abstract

**Aims:**

CircRNAs could regulate macrophage pyroptosis, which has the potential in promoting the synergistic effect of inflammation and matrix metalloproteinase (MMP) activity in abdominal aortic aneurysm (AAA). But the roles of circRNAs in modulating macrophage pyroptosis in the AAA remain unknown. This study explored the contribution to AAA of circHipk3, which was macrophage pyroptosis promoter, and the underlying mechanism.

**Methods and Results:**

CircHipk3 was markedly upregulated in aortic aneurysms compared with that in normal arteries. In mice treated with circHipk3 contributed to macrophage pyroptosis, subsequently promoting the synergistic effect of inflammation and MMP synthesis, and significantly accelerated angiotensin (Ang) II‐ and porcine pancreatic elastase (PPE)‐induced AAA formation. Mechanically, chromatin isolation by RNA purification (ChIRP) indicated that circHipk3 facilitated macrophage pyroptosis by interaction with Stat3, increase the NLRP3 level in the aorta, and by binding Snd1 to promote Ptbp1 mRNA degradation to inhibit autophagy. Therefore, our study revealed the important role of circHipk3 in macrophage pyroptosis and thus significantly improved the outcome of AAA.

**Conclusions:**

CircHipk3 serves a dual role in augmenting macrophage pyroptosis by interaction with Stat3, increase the NLRP3 level, and by binding Snd1 to promote Ptbp1 mRNA degradation to inhibit autophagy, thereby inducing aneurysm formation and progression.

**Key points:**

- CircHipk3 is significantly upregulated in abdominal aortic aneurysms (AAA) compared to normal arteries, contributing to macrophage pyroptosis.
- CircHipk3 promotes the synergistic effect of inflammation and matrix metalloproteinase (MMP) activity, accelerating Angiotensin II‐ and porcine pancreatic elastase‐induced AAA formation in mice. Mechanistically, CircHipk3 interacts with Stat3 to elevate NLRP3 levels and binds Snd1 to promote Ptbp1 mRNA degradation, inhibiting autophagy.
- CircHipk3's dual role in enhancing NLRP3 inflammasome activation and inhibiting autophagy makes it a critical regulator in AAA development and rupture.
- Targeting CircHipk3 may offer a novel therapeutic strategy to prevent pyroptosis and AAA development, positioning it as a potential treatment target.

## Graphical abstract

1. CircHipk3‐mediated macrophage pyroptosis acts as a critical upstream regulator of inflammatory cytokines and matrix metalloproteinases (MMPs), significantly contributing to abdominal aortic aneurysm (AAA) formation and rupture.
2. CircHipk3 enhances NLRP3 inflammasome activation by interacting with Stat3 and degrading Ptbp1 mRNA, thereby inhibiting autophagy.
3. Targeting CircHipk3 could offer a novel therapeutic strategy for preventing pyroptosis and AAA development, highlighting its potential as a treatment target.

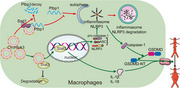

## 1 INTRODUCTION

Abdominal aortic aneurysm (AAA) is a chronic inflammatory disease that causes focal dilation exceeding 50% of the normal diameter in the abdominal aorta, potentially resulting in vascular rupture and high morbidity and mortality rates.^1^ The inflammation process is crucial for the development and progression of aneurysm, as it occurs early, is sustained throughout the course of AAA, and enhances the activity of matrix metalloproteinases (MMPs) and apoptosis of smooth muscle cells.^1^ Pyroptosis is a pro‐inflammatory programmed cell death process and could be both the cause and the consequence of inflammation, forming positive feedback to aggravate inflammation.^2^ Pyroptosis induced cell swelling and rupture of the membrane, causing massive leakage of inflammatory cytokine IL‐1β and IL‐18, high mobility group box 1 (HMGB1) and several S100 proteins, which in turn recruited inflammatory cells and increase inflammatory cell infiltration.^3^ Pyroptotic cells could release apoptosis‐associated speck‐like protein specks (ASC specks) that are rich in inflammasome signals, which could then amplify the inflammatory response.^4^ Pro‐inflammatory cytokines such as tumour necrosis factor alpha (TNF‐α) also activated caspase8‐dependent gasdermin D processing to induce pyroptosis.^5^ More important, previous studies found that pyroptosis marker (NLRP3, CASP1) was increased in AAA^6^ and pyroptosis could promote a variety of important pathological processes of AAA (such as smooth muscle cell phenotype transformation,^7^ T cell infiltration^8^) to induce the formation of AAA, which indicated pyroptosis played a critical role in mediating AAA formation. We believed that pyroptosis serves as a trigger for inflammation, thereby resulting in the formation of AAA. Thus, inhibiting pyroptosis might serve to mitigate the inflammatory response, ultimately suppressing AAA formation.

Macrophages are considered as the primary sources of interleukin (IL)‐1β and IL‐18,^9^, ^10^ which are important pyroptosis‐related cytokines. Additionally, macrophage NLRP3‐CASP1 cascade, activator of pyroptosis, trigger activation of MMP by cleaving its N‐terminal inhibitory domain to initiate AAA formation.^11^ Hence, macrophage pyroptosis could aggravate inflammatory responses and MMP activity to induce AAA formation. Inhibition of macrophage pyroptosis might be a promising approach for preventing the AAA formation. In addition, previous study has proven that gene interventions such as the encoding gene LMP7 downregulation could prevent AAA formation by attenuating macrophage pyroptosis.^12^ Although most encoding genes are expressed across a wide range of cell and tissue types, intervening with them may elicit systemic side effects.^13^ CircRNAs are characterised by highly tissue specific regulated and structural stability,^14^ which give them the potential to delay the AAA formation with minimal systemic adverse effects. However, whether CircRNAs regulated AAA formation by modulating macrophage pyroptosis to suppress inflammatory responses and MMP activity have not been proven.

Emerging evidence shows that circHipk3 involved in pyroptosis of many types of cells, including macrophages, promoted the occurrence of various inflammatory diseases, such as septic acute kidney injury,^15^ acute pancreatitis^16^ and gouty arthritis.^17^ In addition, the sequence of circHipk3 has been shown to be complementary to the seed sequence of miRNA‐124,^15^ miR‐193a‐5p,^16^ miR‐192 and miR‐561,^17^ which exertes significant effects on pyroptosis. Moreover, circHipk3 could promote the synthesis of MMP^18^ and the secretion of inflammatory cytokines IL‐1β, IL‐6, IL‐8 and TNF‐α^16^ in airway smooth muscle and acinar cells, respectively. We therefore hypothesised that circHipk3 aggravate inflammatory cytokines secretion and gelatinolytic enzyme activity by augmenting macrophage pyroptosis to promotes AAA formation.

Consistent with this hypothesis, utilising single‐cell sequencing, we identified the pyroptosis is in large part mediated by macrophages in the course of AAA. Using human specimens validation, Ang II‐ and porcine pancreatic elastase (PPE)‐induced AAA models, dual validation of knockdown and overexpression, we innovative found circHipk3‐mediated macrophage pyroptosis promote the synergistic effect of inflammation and MMP synthesis to exacerbate AAA pathology. Moreover, we conducted dual validation of chromatin isolation by RNA purification (ChIRP) and RNA immunoprecipitation (RIP) assays, combined with microarray technology to search circHipk3 downstream target proteins Stat3, which previously reported to bind to the NLRP3 promoter and enhanced NLRP3 transcription to promote macrophage pyroptosis. Additional, we also found circHipk3 bound Snd1 to promote Ptbp1 mRNA degradation to inhibit autophagy and subsequently reduce the degradation of NLRP3 inflammasome. Thus, inhibition of macrophage pyroptosis by intervening circHipk3 retarded inflammatory cytokines secretion and gelatinolytic enzyme activity, and was a promising approach for mitigating AAA formation.

## 2 MATERIALS AND METHODS

### 2.1 Ethics approval

The human tissue research protocols adhered to the ethical standards outlined in the Declaration of Helsinki and received approval from the Institutional Review Boards of Nanfang Hospital. Participants provided written informed consent prior to their inclusion in the study. The animal research protocols were sanctioned by the Animal Research Committee at Southern Medical University, and all experimental procedures were conducted in strict compliance with the Guide for the Care and Use of Laboratory Animals, as published by the National Institutes of Health (Eighth Edition).

### 2.2 Human aortic samples

In this study, the human tissue protocol was meticulously designed in accordance with the ethical principles enshrined in the Declaration of Helsinki, which serves as a foundational document in international medical ethics. The utilisation of human aortic samples for research purposes was subject to stringent evaluation by the Research Ethics Committees of Nanfang Hospital. Upon thorough review, the protocol was granted ethical approval, ensuring the protection of human subjects and the integrity of the research. Samples of AAA were procured from patients who underwent open surgical repair, while control samples were procured from the adjacent non‐aneurysmal aortic tissue of the same patients, who provided informed consent prior to enrolment. The obtained samples were preserved in a cryogenic environment, either in either in a −80°C freezer or liquid nitrogen for long‐term storage. The demographic and clinical characteristics of the subjects with AAA, encompassing age, sex, smoking status and hypertension, are systematically detailed in Table S1 of the document.

### 2.3 Data sources

To delve into the pathophysiology of AAA, we have accessed relevant public datasets from the Gene Expression Omnibus (GEO), a repository maintained by the National Center for Biotechnology Information (NCBI). The datasets are readily accessible through the GEO portal at http://www.ncbi.nlm.nih.gov/geo/. The repository's open access policy negates the need for patient consent or ethical approval for the utilisation of the data. The publicly accessible single‐cell RNA sequencing (scRNA‐seq) dataset, encompassing infrarenal abdominal aortas (IAAs) from C57BL/6J mice at 14 days post‐periadventitial elastase‐induced AAA or 14 days post‐periadventitial heat‐inactivated elastase incubation, was obtained from the GEO database (GSE152583). In the specific dataset GSE51227, which we utilised for our study, mRNA expression profiles from five AAA patient samples and five control samples from healthy individuals were included.

#### 2.3.1 Quality control and normalisation

The single‐cell gene expression matrix was analysed using Seurat (v4.0.2), an R package designed for scRNA‐seq data analysis. To ensure data quality, cells with fewer than 200 expressed genes or with mitochondrial read counts exceeding 5% were excluded. Genes expressed in fewer than three cells were also filtered out. The count matrix of the remaining cells was then normalised using Seurat's normalisation function. The top 2000 highly variable genes were identified with the ‘vst’ method through the FindVariableFeatures function, and the normalised data were further processed with the ScaleData function.

#### 2.3.2 Unsupervised clustering analysis and cell annotation

The highly variable genes were subjected to principal component analysis (PCA). To correct for batch effects and integrate the scRNA‐seq data, the Harmony R package was employed, which aligns cells into a shared embedding that groups them by cell type rather than by dataset‐specific conditions. Unsupervised cell clustering was performed using the top 20 principal components with the Seurat functions FindNeighbors and FindClusters. For visualisation, cells were projected into a two‐dimensional (2D) space using Uniform Manifold Approximation and Projection (UMAP). To pinpoint genes specific to each cluster, we used the FindAllMarkers function, applying a Wilcoxon rank‐sum test. Cell types were manually annotated based on highly specific marker genes associated with each cluster.

### 2.4 Experimental animals and AAA models

The animal protocol in this study was subjected to a comprehensive and stringent review process, culminating in approval by the Animal Research Committee of Southern Medical University. This approval was contingent upon the adherence to the highest standards of animal welfare and experimental conduct. In addition, the care and handling of the animals, as well as the experimental procedures, were conducted in strict accordance with the ‘Guide for the Care and Use of Laboratory Animals’ (Eighth Edition), as published by the National Institutes of Health. This guide is a benchmark for ethical animal research and ensures the humane treatment of laboratory animals. The mice utilised in the study were housed in a pathogen‐free environment, ensuring their health and the validity of the experimental outcomes. They were provided with a standard diet of chow and access to water ad libitum. The housing conditions were meticulously controlled to maintain a constant temperature of 22°C, relative humidity within the range of 60%–65%. These controlled environmental conditions are essential for the well‐being of the animals and the reproducibility of the experimental results.

#### 2.4.1 Ang II‐induced AAA model

The AAA model was induced by angiotensin II (Ang II) in accordance with previously established methodologies. The study utilised male C57BL/6J mice aged 10–12 weeks and male apolipoprotein E‐deficient (apolipoprotein−/−) mice aged 12–16 weeks. Anaesthesia was induced using 2% isoflurane, and the depth of anaesthesia was meticulously monitored by evaluating the absence of withdrawal reflex to toe pinch and observing the respiratory rate to ensure the well‐being of the animals. Upon achieving an adequate level of anaesthesia, the mice were implanted with either Ang II (A9525, Sigma) or a control solution of normal saline using an implantable osmotic pump (ALZET, Model 2004, DURECT Corporation). The implantation was performed via subcutaneous injection, with the pump delivering the solution at a constant rate of 1 mg/kg/min over a period of 28 days. Surgical incision was made in the subcutaneous tissue at the nape of the neck under anaesthesia, and the micropump was inserted to facilitate continuous delivery of the Ang II or saline. Post‐surgical care was provided to ensure the recovery and monitoring of the animals. At the conclusion of the 28‐day experimental period, the animals were humanely euthanised, and their aortas were harvested for subsequent histological examination. The aortic tissues were subjected to immunohistochemical analysis, Elastic van Gieson (EVG) staining to assess the structural integrity of the aortic wall, and Western blotting to evaluate protein expression levels, providing a comprehensive assessment of the effects of Ang II on the development of AAA in the experimental mice.

#### 2.4.2 PPE‐induced AAA model

Male C57BL/6J mice, aged 10–12 weeks, were employed in this study. The mice were subjected to anaesthesia using 2% isoflurane, and the depth of anaesthesia was closely monitored by assessing the absence of a toe pinch reflex and observing the respiratory rate to ensure proper sedation levels. Surgical procedures were conducted under aseptic conditions, with particular care taken to meticulously dissect the abdominal aorta, separating it from surrounding tissues. This dissection was performed from the subdiaphragmatic renal artery to the iliac artery bifurcation. The internal diameter of the aorta was measured thrice using a video microscope to ensure accuracy and reproducibility. Following the measurement, a small gelatin sponge saturated with 30 µmol/L of the pharmacological agent PPE was applied circumferentially around the peri‐aortic blood vessels for a duration of 10 min. This treatment was compared against a control group, where a gelatin sponge soaked in a .9% sodium chloride solution was applied for an equivalent period. After the application, the aorta was thoroughly rinsed with .9% sterile saline to remove any residual treatment solution. The surgical incision was then closed with sutures. Post‐operative care was provided to the animals to facilitate recovery. Two weeks post‐treatment, the animals were humanely euthanised, and their aortas were harvested for detailed histological examination. Tissue samples were subjected to immunohistochemical analysis to detect specific cellular markers, EVG staining to evaluate the structural integrity and composition of the aortic wall, and Western blotting to assess protein expression levels.

### 2.5 Cell culture and transfection/treatment

To procure bone marrow‐derived macrophages (BMDMs), male C57BL/6J mice were euthanised via cervical dislocation and disinfected with 75% ethanol. The femora were excised, and the marrow was flushed, yielding a cell suspension. Following centrifugation at 310 × *g* for 5 min, erythrocytes were lysed using red blood cell lysing buffer. The resultant cell population was cultured in RPMI 1640 medium supplemented with 10% foetal bovine serum (FBS), 15% L929‐conditioned medium, 30 U/mL penicillin and 30 µg/mL streptomycin to induce macrophage differentiation. On day 5, the medium was replenished, and the BMDMs were collected on day 7, quantified using an automated cell counter, and plated in 6‐well plates for subsequent analysis.

For the transfection of small interfering RNA (siRNA), cells were initially plated in 6‐well plates to achieve a density of 10^6^ cells/mL. Following this, the cells underwent serum deprivation to synchronise their growth phase. They were then re‐transfected with siRNA targeting circHipk3 (mmu_circ_0001052), sourced from the circBase database, utilising LipoFiter™ lipofection reagent (Invitrogen, Hanbio Biotechnology Co., Ltd). Post an 8‐h incubation, the medium was exchanged for Dulbecco's modified Eagle medium (DMEM).

To induce pyroptosis in macrophages, we employed lipopolysaccharide (LPS) at a concentration of 500 ng/mL to stimulate the macrophages for a period of 24 h.

### 2.6 Transmission electron microscope observation

BMDMs were inoculated into 10 cm culture plates and cultured for 24 h. They were then exposed to the aforementioned different stimuli and interventions as per the previously described method for an additional 24 h. Subsequently, the cells were collected and fixed with an electron microscope fixative solution. The integrity of the BMDM cell membranes and the morphology of the cellular organelles were observed using a transmission electron microscope (FEI Tecnai G2 20, TWIN).

### 2.7 Real‐time PCR

In vitro cultured BMDMs were employed for the isolation of total RNA using TRIzol reagent (Invitrogen), following the protocol provided by the manufacturer. Thereafter, reverse transcription was performed to convert the isolated total RNA into complementary DNA (cDNA) using a reverse transcriptase enzyme (Takara Biotechnology). Quantitative real‐time polymerase chain reaction (qRT‐PCR) was conducted to quantify the expression of the gene of interest, employing the SYBR Green RT‐PCR kit (Takara Biotechnology) and the Light Cycler 480 II system (Roche Diagnostics), as per established methodologies. Glyceraldehyde‐3‐phosphate dehydrogenase (GAPDH) served as an endogenous control to normalise the data, applying the 2−ΔΔCt method for relative quantification. The sequences of the qRT‐PCR primers, which were synthesised by Qingke Biotechnology Co., Ltd., are detailed in Table S2.

### 2.8 Infection of adeno‐associated virus 9 in mice

#### 2.8.1 Ang II‐induced AAA model

Adeno‐associated virus 9 (AAV9) vectors for murine circHipk3 overexpression, circHipk3 knockdown and their respective controls were synthesised by Obio Technology. In the context of in vivo investigations, both ApoE−/− and C57BL/6J mice were administered 1 × 10^11^ viral genome particles of AAV9 via tail vein injection, or directly into the abdominal aorta. Subsequent to the viral administration, a 30‐day period was allowed for transduction, after which the mice were subjected to AAA model induction.

### 2.9 Local delivery of ADV

All of the murine circHipk3‐overexpression adenovirus (ADV), circHipk3‐knockdown ADV and corresponding control ADV were synthesised by Obio Technology.

To enable local delivery of ADV to the aortic wall and avoid potential systemic side effects, we used a local delivery model established by F‐127 Pluronic gel (Sigma).

#### 2.9.1 PPE‐induced AAA model

The abdomen was incised to fully expose about 5–8 mm of the lower renal abdominal aorta, and the connective adipose tissue around the aorta was cleaned, infiltrated with a small piece of gelatin sponge, and applied externally with elastase for 10 min, rinsed three times with normal saline and kept at 4°C. Next, a 1:1 volume mixture of the viral solution containing 1 × 10^11^ viral genome particles and 20% F‐127 gel supplemented with .25% trypsin was prepared, and gently applied around the exposed section of the abdominal aorta.

### 2.10 Protein extraction and Western blotting

Protein extraction from both cellular and aortic tissue samples was performed using radioimmunoprecipitation assay (RIPA) buffer, composed of 25 mM Tris–HCl at pH 7.6, 150 mM NaCl, 1% NP‐40 and 1% sodium dodecyl sulphate (SDS). Protein concentrations were quantified using the Bicinchoninic Acid (BCA) protein assay kit (Beyotime, P0010) according to the manufacturer's protocol. Following quantification, proteins were denatured at 100°C for 10 min and resolved on SDS‐polyacrylamide gel electrophoresis (PAGE) gels with varying percentages (8%, 10% or 12%) depending on the molecular weight of the target proteins. After electrophoresis, proteins were transferred onto nitrocellulose membranes. Membranes were blocked with 5% bovine serum albumin (BSA) at room temperature for 1 h to reduce non‐specific binding. Primary antibodies were then applied and incubated overnight at 4°C. After three washes with Tris‐buffered saline containing Tween‐20 (TBST) for 10 min each, membranes were incubated with horseradish peroxidase (HRP)‐conjugated secondary antibodies for 1 h at room temperature. Following another series of three washes with TBST, protein bands were visualised using an enhanced chemiluminescence (ECL) substrate (ECL Advance; no. RPN2235, GE Healthcare Life Sciences). Signal detection and image capture were accomplished using a ChemiDoc imaging system (Bio‐Rad Laboratories). Band intensities were quantified using ImageJ software (National Institutes of Health), with GAPDH as an endogenous loading control to normalise protein expression levels. Details regarding the primary antibodies used are provided in Table S3.

### 2.11 Histological analyses

Upon necropsy, the aortic segment extending from the aortic arch to the iliac bifurcation was dissected. The aorta was perfused with physiological saline, fixed in 4% paraformaldehyde in phosphate‐buffered saline (PBS) for 5 min and then excised. Following this, the tissue was maintained in the fixative for 24 h prior to paraffin embedding. Serial sections, 5 µm in thickness, were generated at 500 µm intervals. A minimum of 10 sections per animal were subjected to immunohistochemical analysis.

### 2.12 Aneurysm quantification

All mice that survived until the endpoint of the study were humanely euthanised, and a laparotomy was performed to ascertain the presence of AAA. An incision through the abdominal wall was followed by the infusion of 10 mL of PBS into the left ventricle, which was then drained from the transected right atrium. Subsequently, the aorta was imaged. The suprarenal aortic segment was delineated as the tract beneath the terminal intercostal arteries and superior to the origin of the right renal artery. To objectively measure AAA dimensions, the maximal transverse diameter of the abdominal aorta was determined using Image‐Pro Plus software (Media Cybernetics). The diagnostic criterion for aneurysm formation was a dilation exceeding 50% of the baseline aortic diameter as observed in control animals, aligning with the clinical definition used for humans. In cases where mice succumbed prior to scheduled euthanasia, a post‐mortem examination was conducted to ascertain aortic rupture. Rupture was confirmed by the presence of haemoperitoneum or haemothorax. Mice that died from aortic rupture were included solely in the analysis of mortality and rupture rates, and were not considered in other experimental assessments. The evaluation of AAA was conducted by investigators who were unaware of the treatment allocation, ensuring the objectivity of the study outcomes.

### 2.13 Immunofluorescence staining

Immunofluorescence staining was executed following established protocols. BMDMs were fixed using 4% paraformaldehyde, permeabilised with .5% Triton X‐100 and blocked with 5% BSA in PBS for 60 min. Subsequently, the cells were incubated with primary antibodies specific for CD68, NLRP3 and CASP1 at 4°C for an extended period. After thorough washing with PBS, the cells were treated with Alexa Fluor‐conjugated secondary antibodies designed for maximal excitation at wavelengths of 488 or 594 nm.

For aortic tissue samples, cryosections were fixed in acetone and blocked with 5% bovine serum in PBS for 1 h at ambient temperature. The sections were then exposed to primary antibodies overnight at 4°C. Following PBS washing, the sections were incubated with Alexa Fluor‐tagged secondary antibodies. Nuclear staining was achieved with 4′,6‐diamidino‐2‐phenylindole (DAPI). Imaging was conducted utilising a Leica TCS SP8 confocal fluorescence microscope. Details of the primary antibodies utilised are provided in Table S4.

### 2.14 Immunohistochemical analysis

Aortic tissue samples underwent deparaffinisation to remove paraffin wax, followed by the quenching of endogenous peroxidase activity using a 3% hydrogen peroxide solution to prevent background staining. Non‐specific binding was blocked with 10% bovine serum for an hour. The samples were then incubated with primary antibodies overnight at 4°C to allow for antibody–antigen interaction. After incubation, the samples were treated with biotinylated secondary antibodies for 30 min, followed by incubation with a HRP‐conjugated streptavidin solution to form a complex with the biotinylated secondary antibodies. The colourimetric reaction was developed using diaminobenzidine (DAB) as the chromogen, which produces a brown precipitate at the site of antibody binding. The samples were lightly counterstained with haematoxylin to provide contrast by staining the nuclei blue. As a negative control, male ApoE−/− mice treated with Ang II were used; in these controls, the primary antibody was omitted, and the samples were only exposed to the secondary antibody. It was observed that all negative control samples exhibited negligible staining, confirming the specificity of the primary antibodies used. Details regarding the primary antibodies are presented in Table S5.

### 2.15 RNA‐in situ hybridisation

Human and murine aortic sections on coverslips were fixed with a 4% paraformaldehyde solution and subsequently rinsed with PBS three times to remove any residual fixative. The samples were then permeabilised using a .2% Triton X‐100 solution in PBS to facilitate probe penetration and mixed with a digoxigenin (DIG)‐labelled human circHipk3 probe (RiboBio) for overnight incubation at 37°C. The circHipk3 probe was procured from Asilon Biotechnology Co., Ltd. After the hybridisation period, the samples were stringently washed with serial dilutions of saline‐sodium citrate (SSC) buffer, starting with 2 × SSC, followed by 1 × SSC and finally .5 × SSC, to remove unhybridised probes. The samples were then incubated with an alkaline phosphatase (AP)‐conjugated mouse anti‐DIG antibody (Boster Biotechnology) to bind to the DIG‐labelled probes within the tissue. The in situ hybridisation (ISH) signals for circHipk3 were visualised as brownish‐yellow speckles, indicating the presence and localisation of circHipk3 within the aortic cells. This technique allows for the detection and quantification of circHipk3 expression at the cellular level, providing valuable insights into its distribution and potential functional roles within the aortic tissue.

### 2.16 Ultrasonic imaging

Employing a Vevo 2100 imaging system with a 40 MHz transducer, we acquired 2D Doppler ultrasound images of the murine aorta under 2% isoflurane anaesthesia. Initial ultrasonographic assessments were conducted 1 day preceding the establishment of the experimental model, with follow‐up measurements on days 14 and 28 post‐initiation of Ang II infusion. Aortic lumen diameter during systole was determined thrice in the longitudinal view of the suprarenal abdominal aorta by an investigator blinded to the experimental conditions. Presented data correspond to the diastolic abdominal aortic lumen diameter for each subject.

### 2.17 ChIRP

ChIRP was performed in accordance with the Magna ChIRP RNA Interactome Kit (Millipore) guidelines. In summary, approximately 1 × 10^7^ cells were lysed using the kit's complete lysis buffer. Sonication was employed to fragment the cellular DNA, reducing it to smaller, manageable pieces. The lysate was incubated with biotinylated RNA probes tailored to specifically anneal with circHipk3 RNA sequences. These probes were bifurcated into ‘odd’ and ‘even’ sets, aligning with their ordinal positions. Additionally, non‐specific control probes were designed to target the LacZ gene. Post‐hybridisation, the RNA‐probe complexes were affinity‐purified using streptavidin‐coated magnetic beads. The co‐isolated DNA was extracted from these complexes and underwent qRT‐PCR analysis. This step was critical for evaluating the precise interactions and the relative abundance of chromatin components associated with circHipk3.

### 2.18 Elastic Van Gieson staining

To assess the extent of elastin degradation, 5 µm serial sections were prepared and subjected to EVG staining using the previously described protocol,^1^ and the degree of degradation was determined based on a scale ranging from 1 (indicating no degradation) to 4 (indicating rupture of the aorta or severe rupture of the elastin layer), with scores of 2 and 3 indicating mild and moderate elastin degradation, respectively, and characterised by a few breaks or multiple breaks.

### 2.19 GFP‐RFP‐LC3 assay

The BMDMs were seeded into sterile confocal dishes (20 mm) and cultured for 1 day. The RFP‐GFP‐LC3 virus (Hanbio Biotechnology) was transfected into the cells. After 4 h, the medium was replaced with 1640 culture medium. After an additional 48 h of culture, the cells were fixed with 4% paraformaldehyde for 15 min. Subsequently, the cells were observed and photographed using a confocal microscope (Leica TCS SP8).

### 2.20 Propidium iodide staining

To determine pyroptosis, a double fluorescent staining (CA1120, Solarbio) method utilising Hoechst and propidium iodide (PI) was applied to cultured cells. BMDMs were cultured in 30 mm confocal dishes and stained with a mixture of Hoechst and PI at a 1:1 ratio, resulting in a final concentration of 5 µL/mL for each dye. The culture dish was covered with the staining solution and incubated for 20 min at 4°C before being washed once with PBS. The stained cells were then examined using a confocal microscope (Leica TCS SP8).

### 2.21 TUNEL staining

The One‐step Terminal deoxynucleotidyl transferase dUTP Nick End Labeling (TUNEL) In Situ Apoptosis Kit (Green, FITC; E‐CK‐A320, Elabscience) was utilised for the detection of apoptotic cells in culture. To quantify the total cell nuclei, DAPI staining was employed. Positive staining for apoptosis was visualised using a laser confocal microscope (Leica TCS SP8).

### 2.22 Measurement of inflammatory cytokines

The levels of IL‐1β and IL‐18 in the supernatants of cultured cells were quantified using ELISA kits (E‐EL‐M0037 for IL‐1β and E‐EL‐M0730 for IL‐18, both from Elabscience) following the manufacturer's protocol. The absorbance of each sample was measured at 450 nm using a spectrophotometer to determine the colour development intensity.

### 2.23 Statistical analysis

Statistical analyses were conducted and graphical representations were generated using GraphPad Prism version 8.0.2 software. Quantitative data were presented as the mean ± standard deviation (SD). Prior to statistical testing, all continuous variables underwent assessment for normal distribution. For comparisons between two groups, the independent samples *t*‐test was applied. When comparing multiple groups, a one‐way analysis of variance (ANOVA) was utilised, followed by the Bonferroni post hoc test for specific between‐group comparisons. For data that did not meet the assumptions of normality or equal variances, the non‐parametric Kruskal–Wallis test was employed, complemented by Dunn's multiple comparisons test for post hoc analysis. The occurrence of aneurysms was evaluated using Fisher's exact test, which is appropriate for categorical data with small sample sizes. Fractional elastin degradation, a non‐normally distributed variable, was expressed as the median with interquartile ranges. A *p* value of less than .05 was established as the threshold for statistical significance.

## 3 RESULTS

### 3.1 Elevated expression of pyroptosis markers and increased fibrosis were observed in both human abdominal aortic aneurysm and Ang II‐induced mouse AAA

To determine if pyroptosis plays a role in human and mouse aneurysms, we evaluated markers of pyroptosis including NLRP3, CASP1, GSDMD and IL‐1β, and also analysed MMP2/9 in human aortic aneurysms and mouse AAA. Furthermore, we performed Masson's trichrome staining to observe the condition of collagen fibres. Human tissue and corresponding adjacent normal samples of AAA were taken from patients who underwent AAA resection surgery. Masson's trichrome showed attenuated collagens in the aorta of the patients with AAAs (Supporting Information Figure S1A). qPCR results showed that the expression of pyroptotic markers NLRP3, CASP1 and IL‐1β were substantially upregulated in human AAA (Supporting Information Figure S1B). Western blotting results revealed that NLRP3, MMP2 and MMP9, which induced the degradation of extracellular matrix (ECM), were dramatically increased in the human and mouse aortic aneurysm tissues compared with non‐aneurysmal tissues (Supporting Information Figure S1C). In Ang II‐induced mouse AAA, the results obtained from Masson's trichrome staining, qPCR and Western blotting experiments were consistent with those from human AAA samples (Supporting Information Figure S1D–F). The tendencies of NLRP3, CASP1, GSDMD and IL‐1β presented by immunohistochemical. Immunohistochemistry was used to examine the expression trends of NLRP3, CASP1, GSDMD and IL‐1β in human and mouse AAA samples. The results showed an upregulation in the expression levels of these key proteins related to pyroptosis in both human and mouse AAA (Supporting Information Figure S1G–N). Collectively, the findings suggest a potential association between augmented pyroptosis and the pathological progression of AAAs.

### 3.2 Increased expression of circHipk human and mouse AAA tissues

**FIGURE 1:**
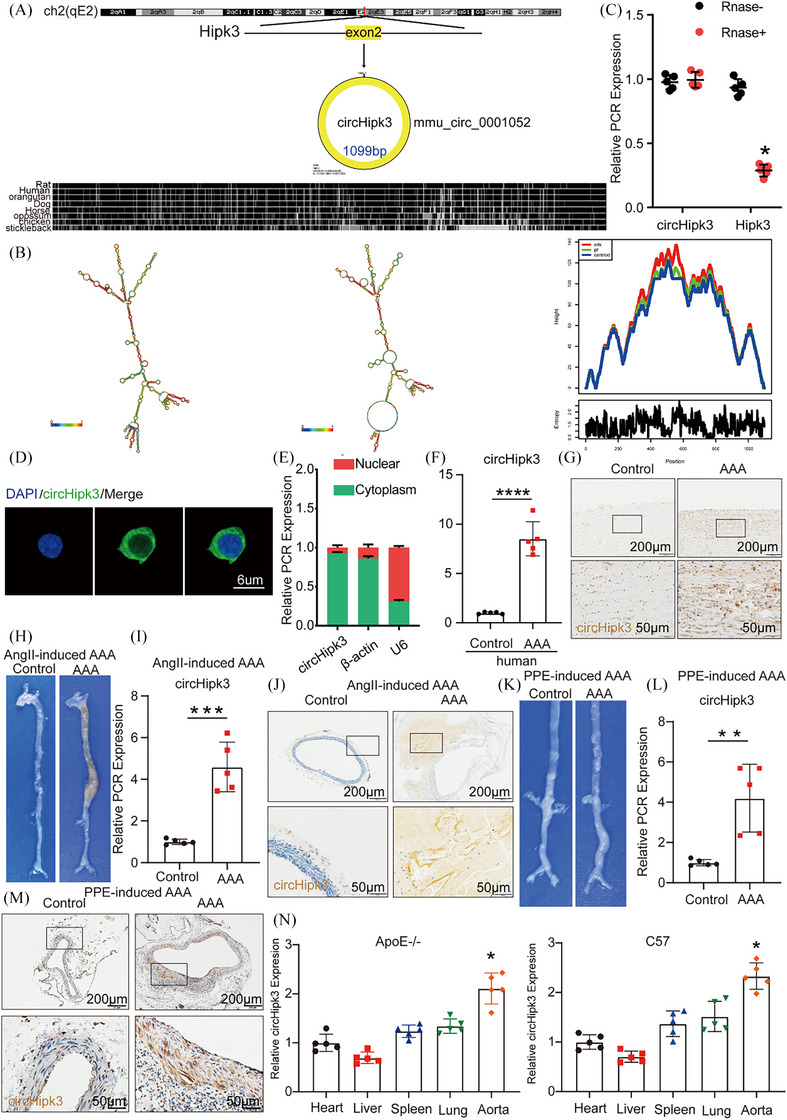
Increased expression of circHipk3 in human and mouse abdominal aortic aneurysm (AAA) tissues. (A) The mouse loci of circHipk3 in the Hipk3 gene. (B) The secondary structure of TPRG1‐AS1 that predicted by RNAfold server. (C) The abundances of circHipk3 and Hipk3 mRNA in macrophage treated with RNase R normalised to those measured in the control. *n* = 5, *p < .05 versus RNase treatment. (D) Fluorescence in situ hybridisation (ISH) to determine the circHipk3 levels in macrophages (bar = 6 µm; *n* = 5). (E) Quantitative real‐time polymerase chain reaction (qRT‐PCR) for the abundance of circHipk3 in either the cytoplasm or nucleus of macrophage. (F) The relative expression of circHipk3 in human AAA tissue and control tissue (qPCR). ****p < .0001; *n* = 5 per group. (G) ISH results of circHipk3 in human abdominal aortic aneurysm tissue and control tissue (*n* = 4, bars: upper 200 µm, lower 50 µm, magnified image). (H) Representative photographs of the macroscopic features from ApoE−/− mice treated with Ang II. (I) Relative expression of circHipk3 in Ang II‐induced mice AAA and NA tissues (qPCR). ***p < .001 versus the NA group, *n* = 5 per group. (J) ISH result of aortic circHipk3 in Ang II‐treated mice (bars: upper 200 µm, lower 50 µm). (K) Representative photographs of the macroscopic features from C57BL/6J treaded with porcine pancreatic elastase (PPE). (L) Relative expression of circHipk3 in PPE‐induced mouse AAA and NA tissues (qPCR). **p < .01 versus the NA group, *n* = 5 per group. (M) ISH result of aortic circHipk3 in PPE‐treated mice (bars: upper 200 µm, lower 50 µm). (N) Relative expression of circHipk3 in heart, liver, spleen, lung and aorta tissues of ApoE−/− mice and C57BL/6J mice. *p < .05, *n* = 5 per group.

CirHipk3, a 1099 bp circular RNA, originates from exon 2 of the Hipk3 gene, located on chromosome 2 (qE2) as referenced in circBank (http://www.circbank.cn/; mmu_circ_0001052; Figure 1A and Supporting Information Figure S5D). Figure 1B illustrates the three‐dimensional structure of circHipk3. Firstly, we confirmed that circHipk3 is more stable than the linear transcript (Figure 1C). Immunofluorescence staining (Figure 1D) was conducted and quantitative real‐time PCR was used to separate cytoplasmic and nuclear RNA from macrophages (Figure 1E) to identify the subcellular distribution of circHipk3. The results suggest that circHipk3 is primarily located in the cytoplasm of macrophages.

Next, to verify that circHipk3 is involved in the pathological process of AAA formation, we examined the expression of circHipk3 in human AAA and mouse AAA. Both qPCR assay and ISH results indicated that the expression of circHipk3 in human AAA tissues was significantly higher than that in adjacent non‐aneurysmal tissues (Figure 1F,G). As previously described, we established the Ang II‐induced mouse AAA model and the PPE‐induced mouse AAA model, as well as controls for both to investigate the different circHipk3 expression levels of the aorta in AAA group and control group mice. In general, more pronounced bulges in the abdominal aorta of the AAA model mice were observed compared with the control group mice, indicating that the well‐accepted types of the AAA model were successfully established (Figure 1H). qPCR and ISH identified that the expression level of circHipk3 in Ang II‐ and PPE‐induced AAA model mice were significantly higher than that in wild‐type control mice (Figure 1H–M). In addition, we detected circHipk3 expression in different organs and tissues of ApoE−/− and C57BL/6J mice and found that circHipk3 was most abundant in the aorta showed by qPCR assays (Figure 1N). We further evaluated the correlation between the expression of circHipk3 and pyroptosis markers NLRP3, CASP1 and IL‐1β in human and mouse AAA tissues. The results showed that in human AAA tissues, the expression of circHipk3 was positively correlated with the expression of NLRP3 and IL‐1β. While there was a trend towards a positive correlation between circHipk3 and CASP1, it did not reach statistical significance (Supporting Information Figure S2A–C). Similarly, in mouse AAA samples, circHipk3 expression was positively correlated with the expression of NLRP3 and CASP1. However, the trend towards a positive correlation between circHipk3 and IL‐1β did not reach statistical significance (Supporting Information Figure S2D–F). This suggests that circHipk3 may be associated with pyroptosis and may potentially be involved in the regulation of the pathological process of AAA. Furthermore, to elucidate the cellular origin of circHipk3 in AAA, we performed immunofluorescence co‐staining of circHipk3 with macrophage marker CD68 and smooth muscle cell marker α‐SM‐actin on human and murine samples. Our findings revealed minimal expression of circHipk3 in normal murine aortas, with high expression of α‐SM‐actin, indicating no co‐localisation, and an absence of CD68 expression (Supporting Information Figure S7D,E). In Ang II‐induced mouse AAA, there was a significant increase in the expression of both circHipk3 and CD68, with substantial co‐localisation, whereas α‐SM‐actin expression was markedly reduced, showing minimal overlap (Supporting Information Figure S7F,G). Similar observations were made in PPE‐induced mouse AAA (Supporting Information Figure S8A,B). Consistent with the murine data, human non‐diseased aorta (NDA) and AAA samples exhibited comparable results (Supporting Information Figure S8C–F). The above results suggest that circHipk3 in murine AAA predominantly originates from macrophages and likely exerts its functions primarily within these cells.

### 3.3 CircHipk3 promotes pyroptosis in macrophages

**FIGURE 2:**
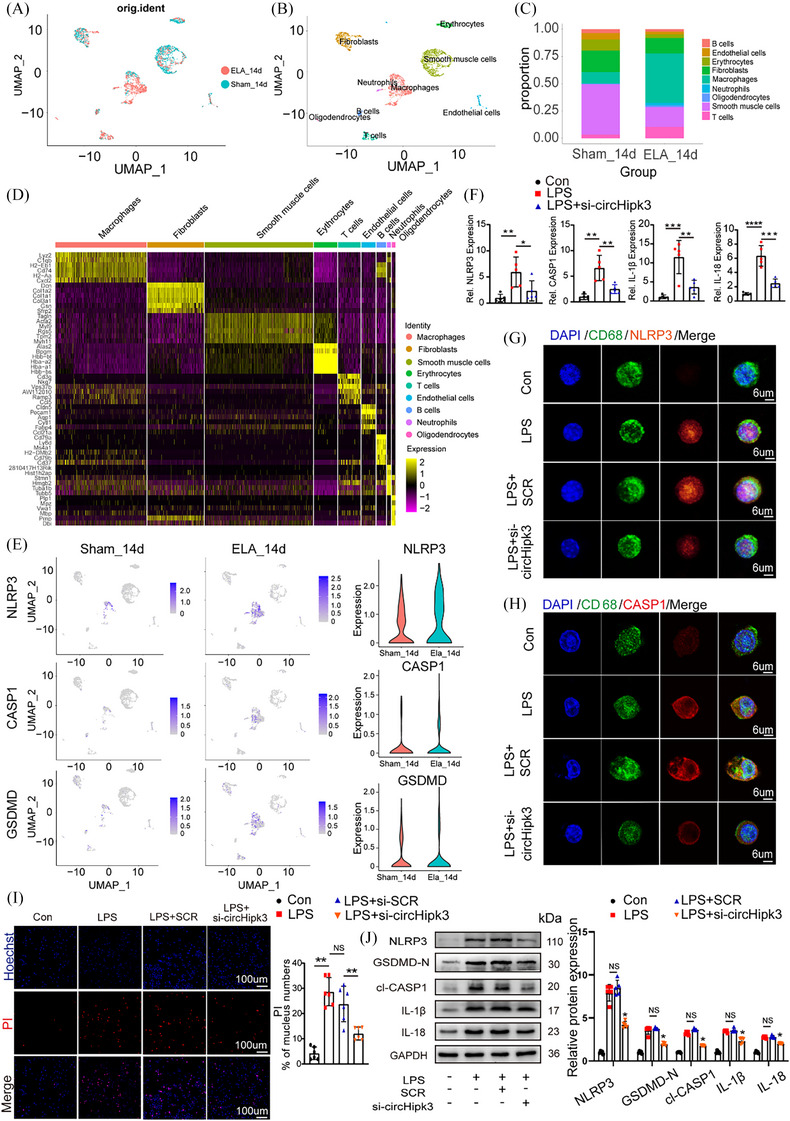
circHipk3 promotes pyroptosis in macrophages. Bone marrow‐derived macrophages (BMDMs) were transfected with either si‐SCR or circHipk3 small interfering RNA fragments for 24 h, followed by treatment with lipopolysaccharide (LPS) at a concentration of 500 ng/mL for an additional 24 h. (A) Uniform Manifold Approximation and Projection (UMAP) visualisation includes 3017 cells derived from the integrated dataset of 14 days post‐periadventitial elastase‐induced (ELA_14d) abdominal aortic aneurysm (AAA) and 14 days post‐periadventitial heat‐inactivated elastase incubation (Sham_14d) abdominal aorta. Colours indicate different conditions. (B) UMAP visualisation of the cell annotation coloured by cell type (*n* = 3017). (C) Proportion of each cell type in different conditions (Sham_14d, ELA_14d). (D) Heatmap of the top five marker genes (by average log [fold change]) expression in nine different cell type. (E) UMAP visualisations depicted the expression profiles of NLRP3, CASP1 and GSDMD in the sham surgery and Elastase‐treated 14‐day groups (left and middle) using a heatmap. The violin plot further illustrated the expression levels of these genes in both the sham surgery and Elastase‐treated 14‐day groups (right). (F) The relative expression of NLRP3, CASP1, IL‐1β and IL‐18 in control group, LPS group and LPS + si‐circHipk3 group (quantitative polymerase chain reaction [qPCR]). *p < .05, **p < .01, ***p < .001, ****p < .0001, *n* = 5 per group. (G) Immunofluorescence staining results for CD68 and NLRP3 in control group, LPS group and LPS + si‐circHipk3 group (bar = 6 µm). (H) Immunofluorescence staining results for CD68 and CASP1 in control group, LPS group and LPS + si‐circHipk3 group (bar = 6 µm). (I) Propidium iodide (PI) staining for macrophage in control group, LPS group and LPS + si‐circHipk3 group (bar = 100 µm). NS, not significant, **p < .001. Quantification of the bands was performed using ImageJ (National Institutes of Health). (J) Western blot analysis of the protein levels of NLRP3, GSDMD‐N, cl‐CASP1, IL‐18, IL‐1β in control group, LPS group, LPS + SCR group and LPS + si‐circHipk3 group. *p < .05, *n* = 5 per group.

In order to examine whether macrophages serve as the leading cell subgroup contributing to the initiation and progression of AAAs, and to determine if cell pyroptosis is predominantly occurring within these macrophages in the context of AAA. We obtained scRNA‐seq data from IAAs of healthy mice and those treated with elastase for 14 days to induce aneurysms (GSE152583, PMID: 32678909). After performing quality control, data integration, dimensionality reduction and clustering of the scRNA‐seq dataset, we manually identified nine distinct cell types of 3017 cells based on marker genes (Figure 2A,B). The results indicate that the percentage of macrophages shows a significant increase in the AAA group compared to the sham group (Figure 2C). The top five specific marker genes for each cell type, identified by average log (fold change), were selected to distinguish each cell type from all others (Figure 2D). In addition, through UMAP and violin plot visualisations, we observed that, compared to the sham group, the pyroptosis marker genes NLRP3, CASP1 and GSDMD were selectively upregulated in the macrophage subsets of the Elastase14d group (Figure 2E). Our results suggest that macrophages, as the main cells of pyroptosis, are involved in the pathological process of AAA pyroptosis. To further explore the physiological role of circHipk3 in macrophages.

In cellular experiments, we induced pyroptosis in macrophages with LPS. First, to further confirm whether circHipk3 plays a role in vascular smooth muscle cells (VSMCs) in addition to macrophages, we examined the localisation of circHipk3 in VSMCs, its impact on pyroptosis, and its relative content. We found that circHipk3 is also primarily located in the cytoplasm (Supporting Information Figure S7A), can promote the expression of the pyroptosis marker NLRP3 in VSMCs (Supporting Information Figure S7B), but compared to macrophages, its expression is lower in VSMCs (Supporting Information Figure S7C), suggesting that circHipk3 does not primarily act in VSMCs. In subsequent cellular experiments, we found that on the basis of LPS‐induced pyroptosis, knockdown of circHipk3 decreased the mRNA levels of NLRP3, CASP1, IL‐1β and IL‐18 (Figure 2F) detected by qPCR. Transmission electron microscopy revealed characteristic features indicative of pyroptosis in macrophages stimulated with LPS, including: (1) cellular swelling and disruption of intracellular organelles, (2) the release of numerous vesicles and cellular debris and (3) chromatin condensation and rupture of the nuclear membrane. These pyroptotic morphological features were attenuated upon knockdown of circHipk3 (Supporting Information Figure S5A). In the chromatin staining experiment (TUNEL), knockdown of circHipk3 resulted in reduced apoptosis in macrophages (Supporting Information Figure S5B,C). As showed by immunofluorescence staining, knockdown of circHipk3 reduced the activation of pyroptosis markers CASP1 and NLRP3 in macrophages after LPS stimulation (Figure 2G,H). Furthermore, knockdown of circHipk3 attenuated rate of macrophage necrosis assessed by PI staining (Figure 2I). Likewise, the similar Western blot results for NLRP3, cl‐CASP1, GSDMD‐N, IL‐1β and IL‐18 was displayed when circHipk3 was knockdowned (Figure 2J). In summary, the results suggest that macrophages play a dominant role in AAA pyroptosis, and circHipk3 can promote the pyroptosis of macrophages.

### 3.4 Knockdown of circHipk3 inhibits Ang II‐induces AAA formation in ApoE−/− mice, and overexpression of circHipk3 promotes AAA formation in Ang II‐induced C57BL/6J mice

**FIGURE 3:**
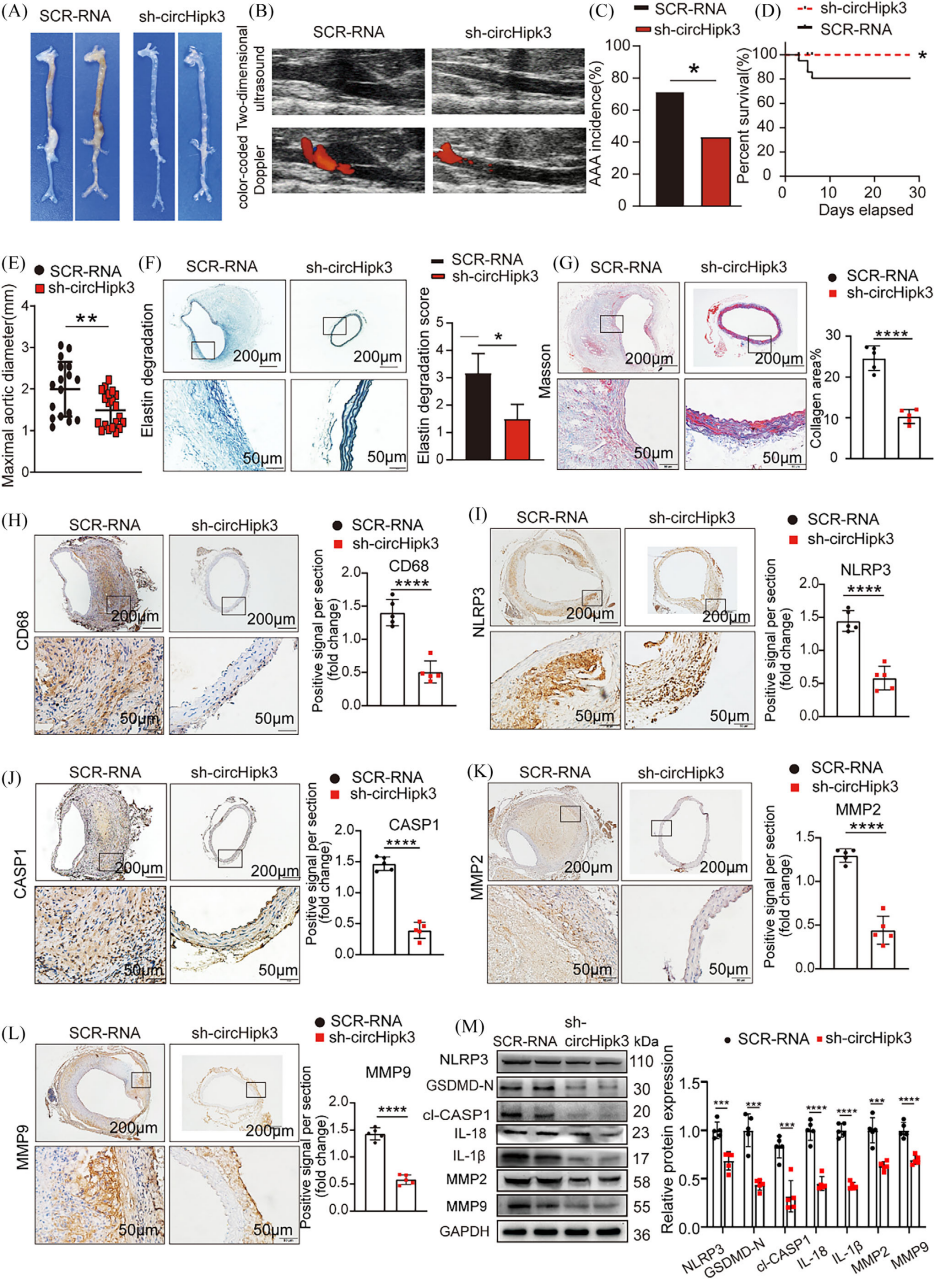
Knockdown of circHipk3 inhibits Ang II‐induced abdominal aortic aneurysm (AAA) formation and reduces the levels of inflammatory molecules associated with pyroptosis and metalloproteinases. (A) Macroscopic images of aortic characteristics of ApoE−/− mice treated with Ang II and circHipk3 knockdown constructs. (B) Two‐dimensional colour‐coded ultrasound imaging of the abdominal aorta from AopE−/− mice in the Scr‐RNA group and the Sh‐circHipk3 group. (C) The incidence of AAA in the Ang II‐induced AAA group or circHipk3 knockdown group, *p < .05, *n* = 20 per group (Fisher's exact test). (D) The survival curve of AAA in the Ang II‐induced AAA group or circHipk3 knockdown group, *p < .05, *n* = 20 per group (log‐rank (Mantel–Cox) test). (E) The maximal abdominal aortic diameter in Ang II‐induced AAA group or circHipk3 knockdown group, **p < .01, *n* = 20 per group (parametric unpaired *t*‐test). (F) Representative elastin staining and elastin degradation scores in the mice aortas of the Ang II‐induced AAA group or circHipk3 knockdown group. Scale bars: upper 200 µm, lower 50 µm, magnified photographs. *p < .05, *n* = 5 per group (non‐parametric analysis of variance [ANOVA] Kruskal–Wallis test with post‐Dunn's multiple comparisons test). (G) Masson's trichrome staining and densitometric analysis in the mice aortas of the Ang II‐induced AAA group or circHipk3 knockdown group (bars: upper 200 µm, lower 50 µm, *n* = 5 per group). ****p < .0001. (H) Representative immunostaining and densitometric analysis of suprarenal aortic CD68 protein expression in the Ang II‐induced AAA group or circHipk3 knockdown group (bars: upper 200 µm, lower 50 µm). ****p < .0001, *n* = 5 per group (parametric unpaired *t*‐test). (I) Representative immunostaining and densitometric analysis of suprarenal aortic NLRP3 protein expression in the Ang II‐induced AAA group or circHipk3 knockdown group (bars: upper 200 µm, lower 50 µm). ****p < .0001, *n* = 5 per group (parametric unpaired *t*‐test). (J) Representative immunostaining and densitometric analysis of suprarenal aortic CASP1 protein expression in the Ang II‐induced AAA group or circHipk3 knockdown group (bars: upper 200 µm, lower 50 µm). ****p < .0001, *n* = 5 per group (parametric unpaired *t*‐test). (K) Representative immunostaining and densitometric analysis of suprarenal aortic MMP2 protein expression in the Ang II‐induced AAA group or circHipk3 knockdown group (bars: upper 200 µm, lower 50 µm). ****p < .0001, *n* = 5 per group (parametric unpaired *t*‐test). (L) Representative immunostaining and densitometric analysis of suprarenal aortic MMP9 protein expression in the Ang II‐induced AAA group or circHipk3 knockdown group (bars: upper 200 µm, lower 50 µm). ****p < .0001, *n* = 5 per group (parametric unpaired *t*‐test). (M) Western blot analysis and densitometric analysis of the protein levels of NLRP3, GSDMD‐N, cl‐CASP1, IL‐18, IL‐1β, MMP2 and MMP9 in the aortas from Ang II‐induced AAA group or circHipk3 knockdown group. (glyceraldehyde‐3‐phosphate dehydrogenase [GAPDH] as the internal reference). ****p < .0001, *n* = 5 per group (parametric unpaired *t*‐test).

**FIGURE 4:**
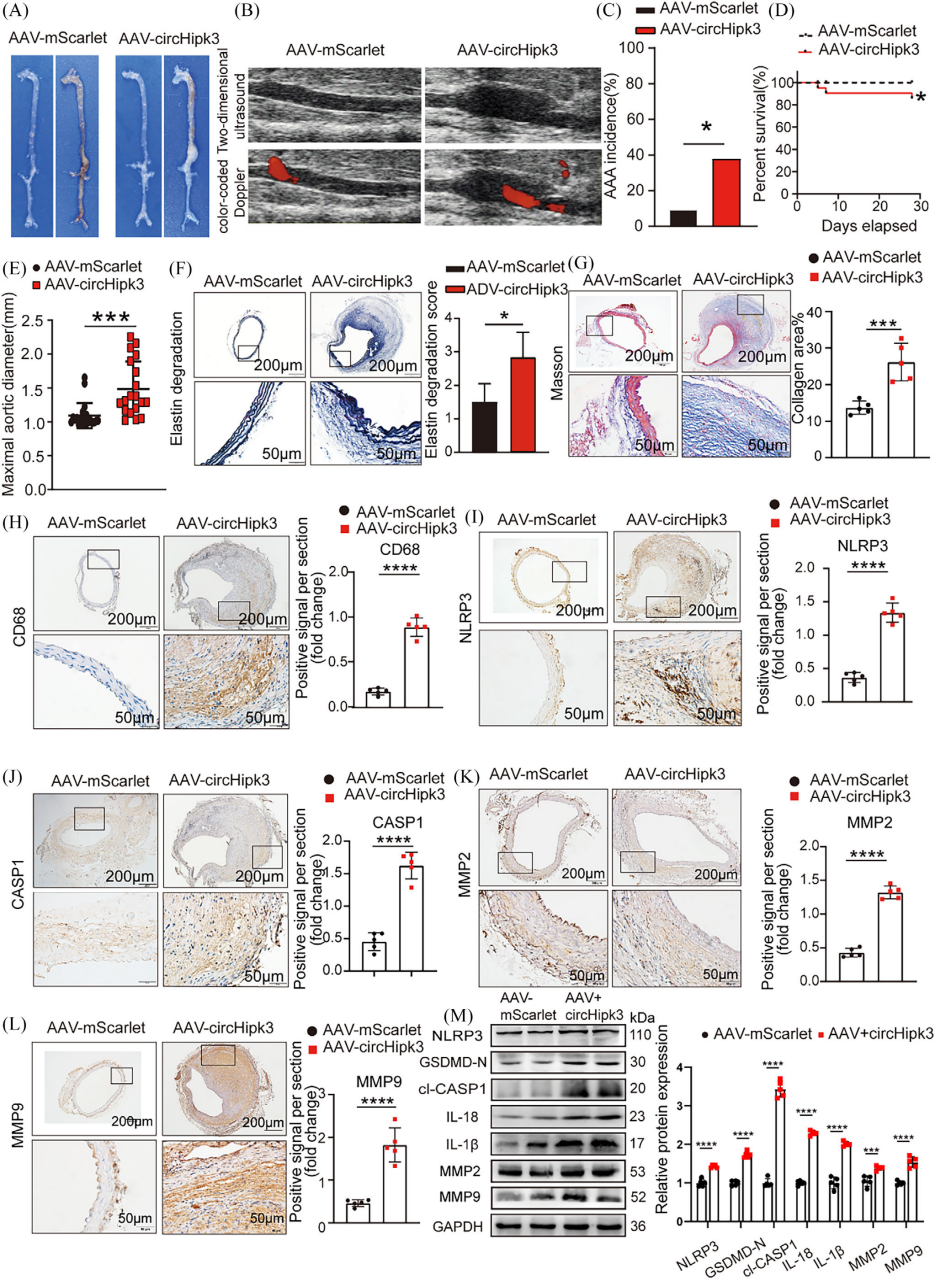
Overexpression of circHipk3 promotes Ang II‐induced abdominal aortic aneurysm (AAA) formation and increases the levels of inflammatory molecules associated with pyroptosis and metalloproteinases. (A) Macroscopic images of aortic characteristics of C57BL/6J mice with Ang II and circHipk3 overexpress constructs. (B) Two‐dimensional colour‐coded ultrasound imaging of the abdominal aorta from C57BL/6J mice in the adenovirus (ADV)‐mScarlet group and the ADV‐circHipk3 group. (C) The incidence of AAA in the Ang II‐induced AAA group or circHipk3 overexpress group *p < .05, *n* = 20 per group (Fisher's exact test). (D) The survival curve of AAA in the Ang II‐induced AAA group or circHipk3 overexpress group,*p < .05, *n* = 20 per group (log‐rank (Mantel–Cox) test). (E) The maximal abdominal aortic diameter in Ang II‐induced AAA group or circHipk3 overexpress group, ***p < .001, *n* = 20 per group (parametric unpaired *t*‐test). (F) Representative elastin staining and elastin degradation scores in the mice aortas of the Ang II‐induced AAA group or circHipk3 overexpress group. Scale bars: upper 200 µm, lower 50 µm, magnified photographs. *p < .05, *n* = 5 per group (non‐parametric analysis of variance [ANOVA] Kruskal–Wallis test with post‐Dunn's multiple comparisons test). (G) Masson's trichrome staining and densitometric analysis in the mice aortas of the Ang II‐induced AAA group or circHipk3 knockdown group (bars: upper 200 µm, lower 50 µm, *n* = 5 per group). ***p < .001. (H) Representative immunostaining and densitometric analysis of suprarenal aortic CD68 protein expression in Ang II‐induced AAA group or circHipk3 overexpress group (bars: upper 200 µm, lower 50 µm). ****p < .0001, *n* = 5 per group (parametric unpaired *t*‐test). (I) Representative immunostaining and densitometric analysis of suprarenal aortic NLRP3 protein expression in Ang II‐induced AAA group or circHipk3 overexpress group (bars: upper 200 µm, lower 50 µm). ****p < .0001, *n* = 5 per group (parametric unpaired *t*‐test). (J) Representative immunostaining and densitometric analysis of suprarenal aorticCASP1 protein expression in Ang II‐induced AAA group or circHipk3 overexpress group (bars: upper 200 µm, lower 50 µm). ****p < .0001, *n* = 5 per group (parametric unpaired *t*‐test). (K) Representative immunostaining and densitometric analysis of suprarenal aortic MMP2 protein expression in Ang II‐induced AAA group or circHipk3 overexpress group (bars: upper 200 µm, lower 50 µm). ****p < .0001, *n* = 5 per group (parametric unpaired *t*‐test). (L) Representative immunostaining and densitometric analysis of suprarenal aortic MMP9 protein expression in Ang II‐induced AAA group or circHipk3 overexpress group (bars: upper 200 µm, lower 50 µm). ****p < .0001, *n* = 5 per group (parametric unpaired *t*‐test). (M) Western blot analysis and densitometric analysis of the protein levels of NLRP3, GSDMD‐N, cl‐CASP1 IL‐18, IL‐1β, MMP2 and MMP9 in the aortas from Ang II‐induced AAA group or circHipk3 overexpress group (glyceraldehyde‐3‐phosphate dehydrogenase [GAPDH] as the internal reference). ***p < .001, ***p < .0001 *n* = 5 per group (parametric unpaired *t*‐test).

To further clarify whether circHipk3 is involved in the occurrence and development of AAA, we used AAV‐9 carrying circHipk3 knockout construct (sh‐circHipk3) or AAV‐9 carrying circHipk3 overexpression construct (AAV‐circHipk3) and the corresponding pseudovirus control group ((AAV)‐SCR‐RNA/AAV‐mScarlet) to perform gain‐ and loss‐of‐function experiments. The in vivo transfection process of AAV is shown in Supporting Information Figure S2G. Immunofluorescence confirmed the successful integration of circHipk3 overexpressed or knockdown constructs into the aortic wall (Supporting Information Figure S3A,B). qPCR confirmed that circHipk3 level was upregulated or downregulated in the aorta of circHipk3‐overexpressed or circHipk3‐knockdowned group mice (Supporting Information Figure S3C,D). As macroscopically observed, the abdominal aorta bulge was significantly reduce in the sh‐circHipk3 mice than in the SCR‐RNA group‐transfected apoE−/− mice (Figure 3A). Similarly, ultrasound imaging also showed the same trend (Figure 3B). Compared with the SCR‐RNA group, knockdown of circHipk3 could decrease the incidence of AAA (Figure 3C), while the rupture rate and maximum aortic diameter of the sh‐circHipk3 group decreased (Figure 3D,E), indicating that circHipk3 can promote AAA formation. We further observed whether circHipk3 induced AAA formation by promoting pyroptosis and ECM degradation. Consistent with our hypothesis, elastic fibre staining (EVG) showed that knockdown of circHipk3 could inhibit Ang II‐induced elastin degradation, and collagen fibres staining (Masson) showed less fibrosis in the sh‐circHipk3 group mice (Figure 3F,G). Immunohistochemical staining showed that in sh‐circHipk3 mice, the expression of macrophage marker CD68, pyroptosis maker NLRP3, CASP1 and metalloproteinases MMP2 and MMP9 was significantly decreased (Figure 3H–L). The Western blot results showed that knockdown of circHipk3 reduced the expression levels of NLRP3, GSDMD‐N, cleaved CASP1, IL‐18, IL‐1β, MMP2 and MMP9 in the mouse aorta (Figure 3M). In contrast, overexpression of circHipk3 in C57BL/6J mice stimulated by Ang II promoted AAA formation in comparison with the AAV‐mScarlet group (Figure 4A), whereas ultrasound imaging (Figure 4B), AAA incidence (Figure 4C), the rupture rate of AAA (Figure 4D) and maximum aortic diameter (Figure 4E), which reflects the severity of AAA lesions was increased in the AAV‐mScarlet group. Consistent with these results, overexpression of circHipk3 promoted Ang II‐induced elastin degradation (Figure 4F), and AAV‐circHipk3 mice showed more fibrosis (Figure 4G). Immunohistochemistry showed that overexpression of circHipk3 upregulated the expression of macrophage marker CD68, pyroptosis maker NLRP3, CASP1, metalloproteinases MMP2 and MMP9 (Figure 4H–L). The Western blot results showed that overexpression of circHipk3 significantly increased the expressions of pyroptosis markers NLRP3, GSDMD‐N, cleaved CASP1, IL‐18, IL‐1β, metalloproteinases MMP2 and MMP9 (Figure 4M). Based on these results, circHipk3 was potent enough to instigate AAA onset by promoting macrophage pyroptosis, subsequently triggering inflammatory cytokines secretion and ECM degradation. To further elucidate the role of circHipk3 in macrophages within AAAs, we generated the HBAAV2/2‐F4/80 virus harbouring the macrophage‐specific promoter driving the expression of circHipk3. After confirming successful transduction of aortic macrophages (Supporting Information Figure S10A) and assessing transduction efficiency (Supporting Information Figure S10B), we observed that overexpression of circHipk3 specifically in macrophages targeted to the abdominal aorta led to an increase in AAA lesion size (Supporting Information Figure S10C), upregulation of NLRP3 expression (Supporting Information Figure S10D). indicating that circHipk3 plays a significant role in macrophage pyroptosis and influences the progression of AAA.

### 3.5 Knockdown of circHipk3 inhibits PPE‐induced AAA formation in C57BL/6J mice and overexpression of circHipk3 promotes PPE‐induced AAA formation in C57BL/6J mice

**FIGURE 5:**
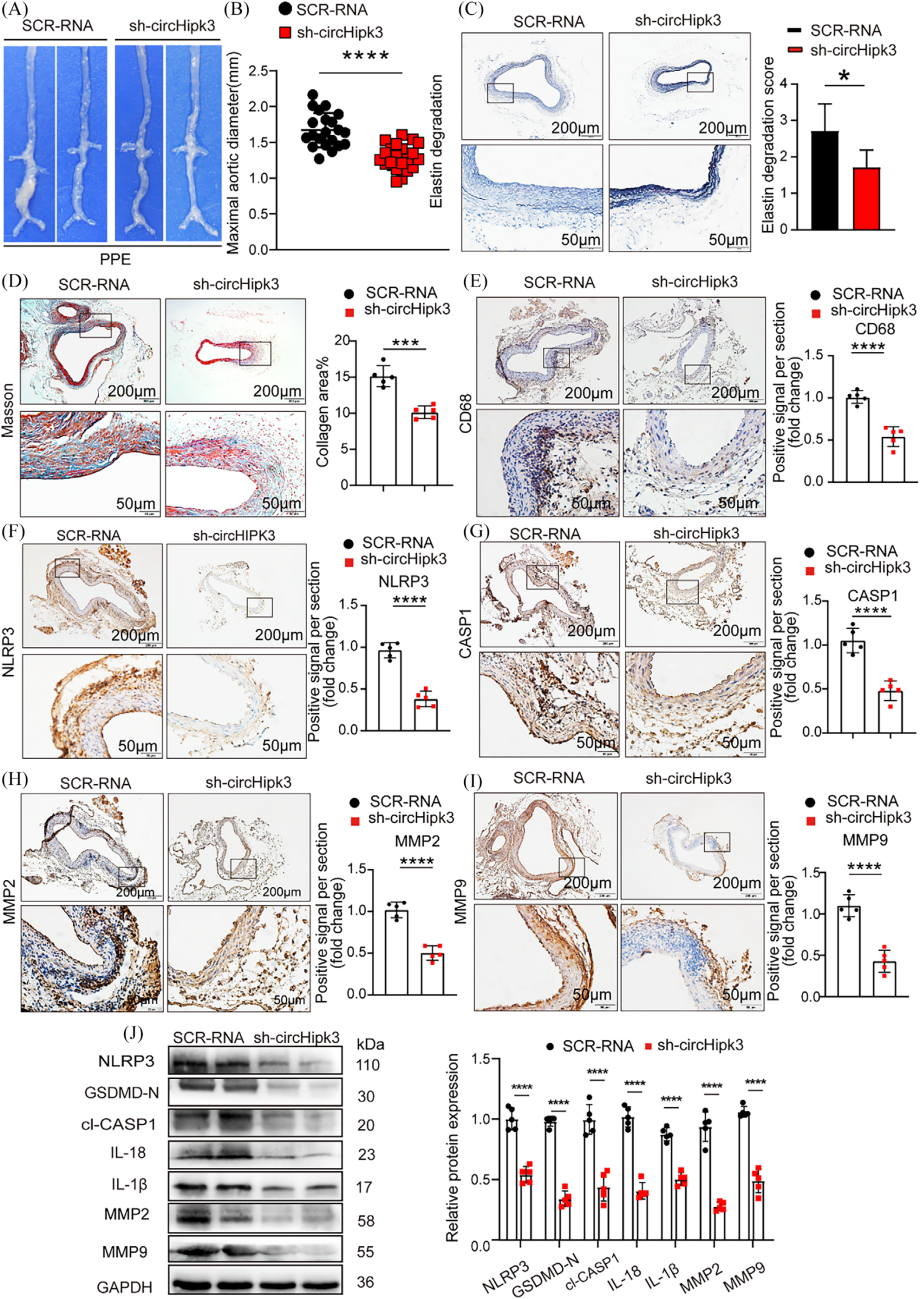
Knockdown of circHipk3 inhibits porcine pancreatic elastase (PPE)‐induced abdominal aortic aneurysm (AAA) formation and reduces the levels of inflammatory molecules associated with pyroptosis and metalloproteinases. (A) Macroscopic images of aortic characteristics of C57BL/6J mice treated with PPE and circHipk3 knockdown constructs. (B) The maximal abdominal aortic diameter in PPE‐induced AAA group or circHipk3 knockdown group, ****p < .0001, *n* = 21 per group (parametric unpaired *t*‐test). (C) Representative elastin staining and elastin degradation scores in the mice aortas of the PPE‐induced AAA group or circHipk3 knockdown group. Scale bars: upper 200 µm, lower 50 µm, magnified photographs. *p < .05, *n* = 5 per group (non‐parametric analysis of variance [ANOVA] Kruskal–Wallis test with post‐Dunn's multiple comparisons test). (D) Masson's trichrome staining and densitometric analysis in the mice aortas of the PPE‐induced AAA group or circHipk3 knockdown group (bars: upper 200 µm, lower 50 µm, ***p < .001, *n* = 5 per group). (E) Representative immunostaining and densitometric analysis of suprarenal aortic CD68 protein expression in PPE‐induced AAA group or circHipk3 knockdown group (bars: upper 200 µm, lower 50 µm). ****p < .0001, *n* = 5 per group (parametric unpaired *t*‐test). (F) Representative immunostaining and densitometric analysis of suprarenal aortic NLRP3 protein expression in PPE‐induced AAA group or circHipk3 knockdown group (bars: upper 200 µm, lower 50 µm). ****p < .0001, *n* = 5 per group (parametric unpaired *t*‐test). (G) Representative immunostaining and densitometric analysis of suprarenal aortic CASP1 protein expression in PPE‐induced AAA group or circHipk3 knockdown group (bars: upper 200 µm, lower 50 µm). ****p < .0001, *n* = 5 per group (parametric unpaired *t*‐test). (H) Representative immunostaining and densitometric analysis of suprarenal aortic MMP2 protein expression in PPE‐induced AAA group or circHipk3 knockdown group (bars: upper 200 µm, lower 50 µm). ****p < .0001, *n* = 5 per group (parametric unpaired *t*‐test). (I) Representative immunostaining and densitometric analysis of suprarenal aortic MMP9 protein expression in PPE‐induced AAA group or circHipk3 knockdown group (bars: upper 200 µm, lower 50 µm). ****p < .0001, *n* = 5 per group (parametric unpaired *t*‐test). (J) Western blot analysis and densitometric analysis of the protein levels of NLRP3, GSDMD‐N, cl‐CASP1, IL‐18, IL‐1β, MMP2 and MMP9 in the aortas from in PPE‐induced AAA group or circHipk3 knockdown group (glyceraldehyde‐3‐phosphate dehydrogenase [GAPDH] as the internal reference). ****p < .0001, *n* = 5 per group (parametric unpaired *t*‐test).

To explore whether the potential effect of circHipk3 on AAA formation is independent of Ang II, and taking into account the possible effects of the utilised animal models and patterns of viral infection, we extended our research to use a PPE‐induced AAA model and ADV intervention techniques to delve deeper into the role of circHipk3 in AAA development. The in vivo transfection process of ADV is shown in Supporting Information Figure S2H. Immunofluorescence confirmed the successful integration of circHipk3 overexpressed or knockdown constructs into the aortic wall (Supporting Information Figure S4A,B). qPCR confirmed that circHipk3 level was upregulated or downregulated in the aorta of circHipk3‐overexpressed or circHipk3‐knockdowned group mice (Supporting Information Figure S4C,D). After PPE was applied to the adventitia of the infrarenal aorta of mice for 2 weeks, the results showed that macroscopically, the mice in the sh‐circHipk3 group exhibited the less pronounced bulge in the abdominal aorta compared with those in the SCR‐RNA group (Figure 5A). In addition, the maximum diameter of the abdominal aorta was decreased in the sh‐circHipk3 group (Figure 5B). Elastic fibre (EVG) staining showed that the degradation of elastin in the sh‐circHipk3 group was milder than that in the SCR‐RNA group (Figure 5C). The result of collagen fibres staining (Masson) showed less fibrosis in the sh‐circHipk3 group mice (Figure 5D). Immunohistochemical staining revealed that the macrophage marker CD68, the pyroptosis marker NLRP3, CASP1 and the metalloproteinases MMP2 and MMP9 displayed identical tendencies to those observed in Ang II‐induced AAA when circHipk3 was knocked down (Figure 5E–I). Additionally, Western blot results showed that knock down of circHipk3 reduced the expressions of NLRP3, GSDMD‐N, cl‐CASP1, IL‐18, IL‐1β, MMP2 and MMP9 in PPE‐induced AAA (Figure 5J).

**FIGURE 6:**
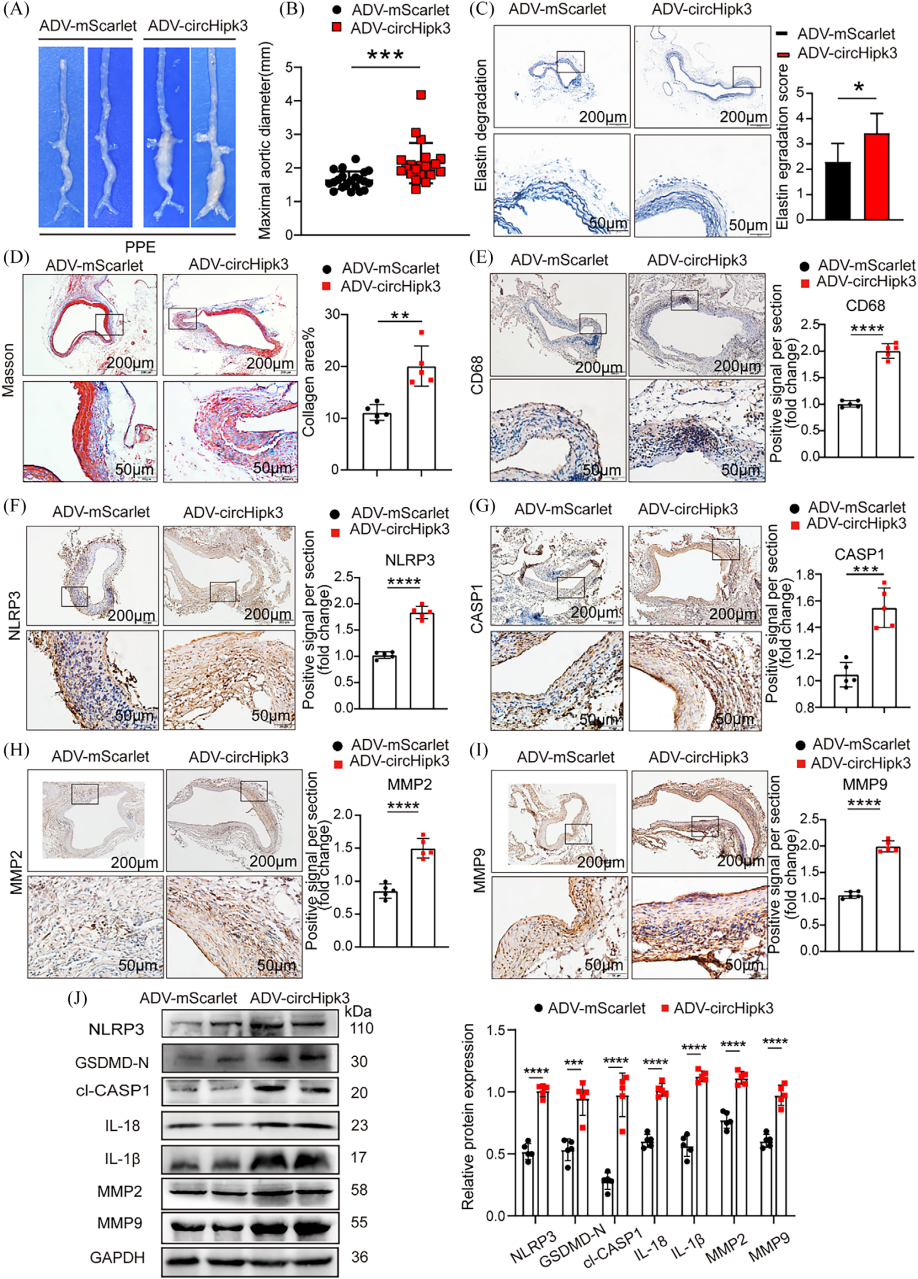
Overexpress of circHipk3 promotes porcine pancreatic elastase (PPE)‐induced abdominal aortic aneurysm (AAA) formation and increases the levels of inflammatory molecules associated with pyroptosis and metalloproteinases. (A) Macroscopic images of aortic characteristics of C57BL/6J mice treated with PPE and circHipk3 overexpress constructs. (B) The maximal abdominal aortic diameter in PPE‐induced AAA group or circHipk3 overexpress group, ***p < .001, *n* = 21 per group (parametric unpaired *t*‐test). (C) Representative elastin staining and elastin degradation scores in the mice aortas of the PPE‐induced AAA group or circHipk3 overexpress group. Scale bars: upper 200 µm, lower 50 µm, magnified photographs. *p < .05, *n* = 5 per group (non‐parametric analysis of variance [ANOVA] Kruskal–Wallis test with post‐Dunn's multiple comparisons test). (E) Representative immunostaining and densitometric analysis of suprarenal aortic CD68 protein expression in PPE‐induced AAA group or circHipk3 overexpress group (bars: upper 200 µm, lower 50 µm). ****p < .0001, *n* = 5 per group (parametric unpaired *t*‐test). (F) Representative immunostaining and densitometric analysis of suprarenal aortic NLRP3 protein expression in PPE‐induced AAA group or circHipk3 overexpress group (bars: upper 200 µm, lower 50 µm). ****p < .0001, *n* = 5 per group (parametric unpaired *t*‐test). (G) Representative immunostaining and densitometric analysis of suprarenal aortic CASP1 protein expression in PPE‐induced AAA group or circHipk3 overexpress group (bars: upper 200 µm, lower 50 µm). ****p < .0001, *n* = 5 per group (parametric unpaired *t*‐test). (H) Representative immunostaining and densitometric analysis of suprarenal aortic MMP2 protein expression in PPE‐induced AAA group or circHipk3 overexpress group (bars: upper 200 µm, lower 50 µm). ****p < .0001, *n* = 5 per group (parametric unpaired *t*‐test). (I) Representative immunostaining and densitometric analysis of suprarenal aortic MMP9 protein expression in PPE‐induced AAA group or circHipk3 overexpress group (bars: upper 200 µm, lower 50 µm). ****p < .0001, *n* = 5 per group (parametric unpaired *t*‐test). (J) Western blot analysis and densitometric analysis of the protein levels of NLRP3, GSDMD‐N, c1cl‐CASP1, IL‐18, IL‐1β, MMP2 and MMP9 in the aortas from PPE‐induced AAA group or circHipk3 overexpress group (glyceraldehyde‐3‐phosphate dehydrogenase [GAPDH] as the internal reference). ****p < .0001, *n* = 5 per group (parametric unpaired *t*‐test).

In contrast, macroscopically, the overexpression of circHipk3 in C57BL/6J mice leads to more prominent AAA dilation under PPE induced compared with ADV‐mScarlet group (Figure 6A). Maximum aortic diameter was increased in ADV‐circHipk3 group (Figure 6B). Moreover, overexpression of circHipk3 promoted PPE‐induced elastin degradation (Figure 6C), and AAV‐circHipk3 mice showed more fibrosis (Figure 6D). Immunohistochemistry showed that overexpression of circHipk3 upregulated the expression of macrophage marker CD68, pyroptosis maker NLRP3, cleaved CASP1, metalloproteinases MMP2 and MMP9 (Figure 6E–I). The Western blot results showed that overexpression of circHipk3 significantly increased the expressions of pyroptosis markers NLRP3, GSDMD‐N, cl‐CASP1, IL‐18, IL‐1β, metalloproteinases MMP2 and MMP9 (Figure 6J). In general, in PPE‐induced AAA, circHipk3 also promotes macrophage pyrolysis, thereby triggering the secretion of inflammatory factors and ECM degradation, accelerating the progression of AAA.

### 3.6 CircHipk3 promotes macrophages pyroptosis through its interaction with Stat3

**FIGURE 7:**
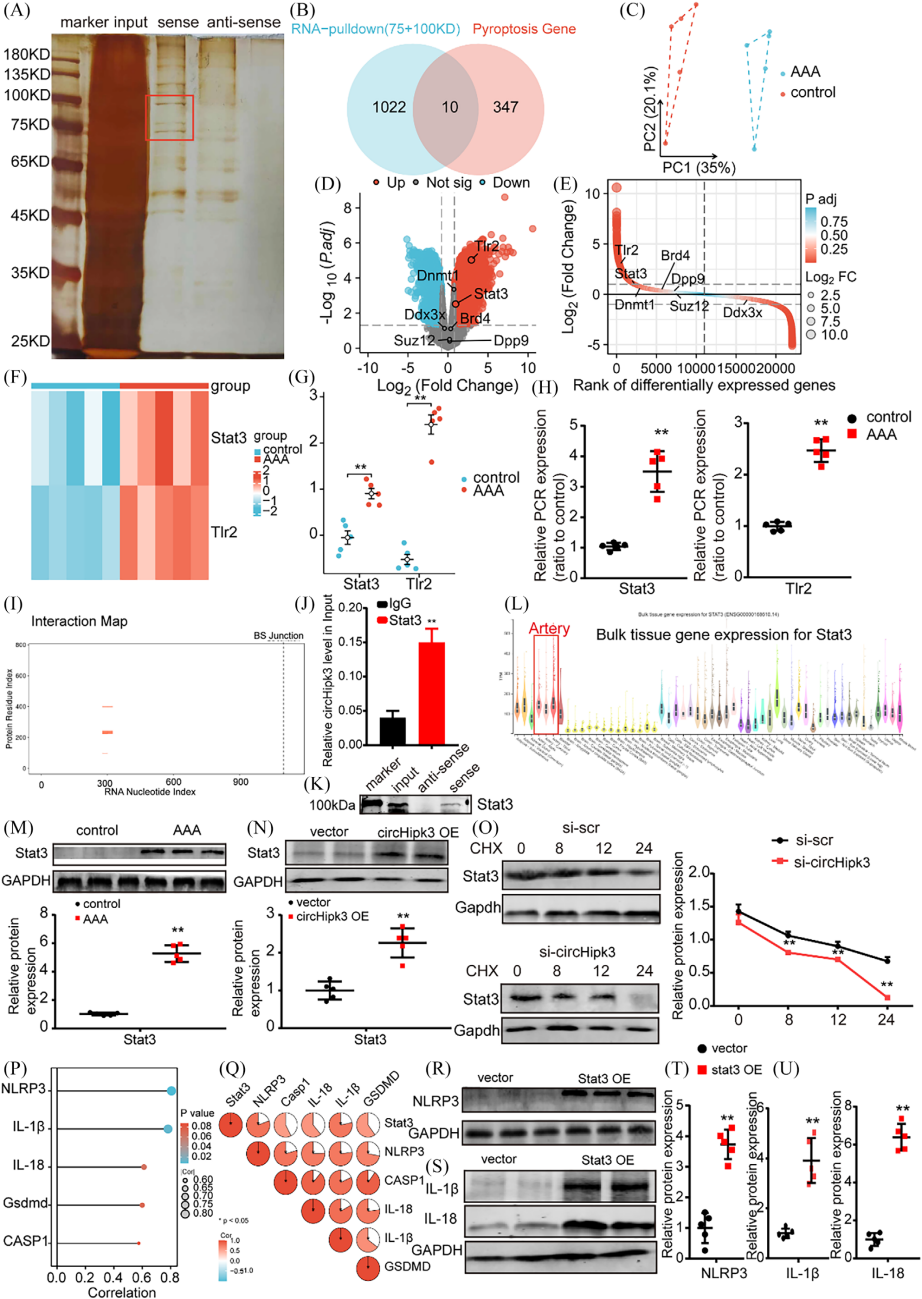
CircHipk3 promotes macrophages pyroptosis through its interaction with Stat3. (A) Sliver‐stained sodium dodecyl sulphate‐polyacrylamide gel electrophoresis gel of circHipk3 and its antisense circRNA‐immunoprecipitated proteins. (B) Venn diagram showing the overlap of circHipk3‐specific binding proteins with pyroptosis‐related genes in genecards. (C) Principal component analysis (PCA) graph in GSE51227 (PMID: 25358394). (D, E) Volcano maps and difference ranking chart of ddx3x, brd4, Stat3 and Tlr2 in GSE51227. (F) Heatmap of ddx3x, brd4, Stat3 and Tlr2 in GSE51227 dataset. (G) Differential expression maps of Stat3 and Tlr2 in GSE51227 (PMID: 25358394). (H) Relative expression of Stat3 and Tlr2 in abdominal aortic aneurysm (AAA) and control (quantitative polymerase chain reaction [qPCR]). **p < .01 versus the control group, *n* = 5 per group. (I) Predicted site map of circHipk3 and Stat3 on the catRAPID website. (J) RNA immunoprecipitation (RIP) was performed using an anti‐Stat3 antibody or a negative control IgG. *n* = 5 per group; **p < .01 versus IgG (Student's *t*‐test). (K) Stat3 was detected by Western blotting. (L) Prediction of the expression of Stat in different tissues via the Genotype‐Tissue Expression Project. (M) Western blot analysis of the protein levels of Stat3 in NA group or AAA group (glyceraldehyde‐3‐phosphate dehydrogenase [GAPDH] as the internal reference). **p < .01, *n* = 5 per group. (N) Western blot analysis of the protein levels of Stat3 protein levels in conditions of circHipk3 overexpression or without (GAPDH as the internal reference). **p < .01, *n* = 5 per group. (O) CircHipk3 was downregulated in macrophages, and the cells were treated with cycloheximide (CHX 50 µg/mL) for the indicated times. Stat3 protein levels were assessed by Western blotting. (P, Q) Correlation analysis between NLRP3, GSDMD, CASP1, IL‐18, IL‐1β and Stat3 in the GSE51227 dataset. (R–U) Western blot analysis and densitometric analysis of the protein levels of NLRP3, IL‐1β and IL‐18 in condition of Stat3 overexpression or without (GAPDH as the internal reference). **p < .01, *n* = 5 per group.

**FIGURE 8:**
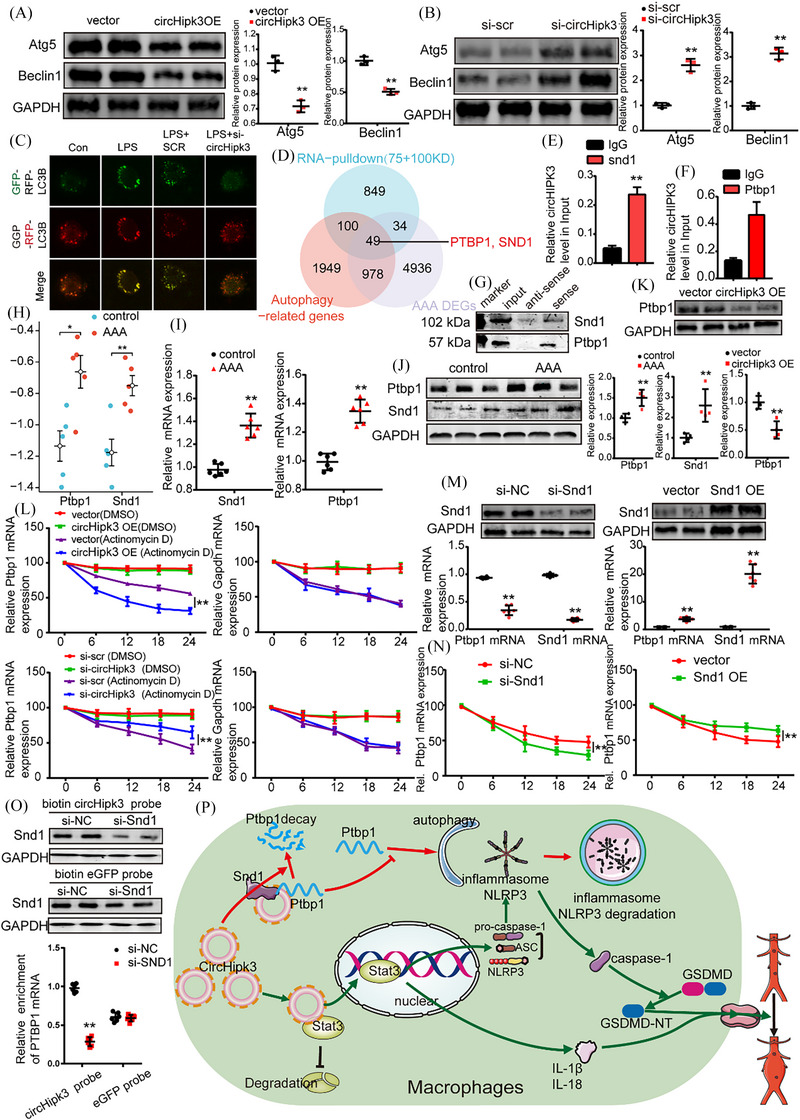
CircHipk3 facilitates macrophage pyroptosis by promoting Ptbp1 mRNA degradation to inhibit autophagy. (A) Western blot analysis was performed to assess the protein levels of ATG5 and Beclin1 in conditions of circHipk3 overexpression compared to control conditions (glyceraldehyde‐3‐phosphate dehydrogenase [GAPDH] as the internal reference). **p < .01, *n* = 5 per group. (B) Western blot analysis was conducted to evaluate the protein levels of ATG5 and Beclin1 under conditions of circHipk3 knockdown compared to control conditions (GAPDH as the internal reference). **p < .01, *n* = 5 per group. (C) Confocal observation results of bone marrow‐derived macrophages (BMDMs) transfected with RFP‐GFP‐LC3 in the control group, lipopolysaccharide (LPS) stimulation group, LPS + SCR group and LPS + circHipk3 knockdown group. (D) Venn diagram showing the overlap of circHipk3‐specific binding proteins with autophagy‐related genes sourced from Genecards and differentially expressed genes (DEGs) obtained from the abdominal aortic aneurysm (AAA) dataset. (E, F) RNA immunoprecipitation (RIP) assays were conducted utilising antibodies against Ptbp1 and Snd1, along with a negative control IgG. *n* = 5 per group; **p < .01 versus IgG (Student's *t*‐test). (G) Ptbp1 and Snd1 were detected by Western blotting. (H) Differential expression maps of Ptbp1 and Snd1 in GSE51227. (I) Quantitative polymerase chain reaction (qPCR) assay of Ptbp1 and Snd1 in AAA and control. **p < .01 versus the control group, *n* = 5 per group. (J) Western blot analysis of the protein levels of Ptbp1 and Snd1 in control group or AAA group (GAPDH as the internal reference). **p < .01, *n* = 5 per group. (K) Western blot analysis of the protein levels of Ptbp1 protein levels in conditions of circHipk3 overexpression or without. **p < .01, *n* = 5 per group. (L) The mRNA levels of Ptbp1 and Gapdh were measured using qPCR following overexpression and knockdown of circHipk3. To inhibit new RNA synthesis, actinomycin (50 uM) was utilised, while dimethylsulfoxide (DMSO) served as the negative control. *p < .05 versus vector (actinomycin D) or shNC (actinomycin D); *n* = 6 per group (one‐way analysis of variance [ANOVA] analysis with Bonferroni's multiple comparison test). (M) Western blot analysis of Snd1 protein level and qPCR analysis of Ptbp1 and Snd1 mRNA levels in conditions of Snd1 overexpression or knockdown (GAPDH as the internal reference). **p < .01, *n* = 5 per group. (N) BMDMs were transfected with either control small interfering RNA (siRNA) or Snd1 siRNA, or mock vector (NC) or pcDNA4.0‐Snd1 plasmid (OE‐Snd1), and subsequently treated with actinomycin D (50 uM) for the specified durations. Data presented represent the mean ± standard deviation (SD) from *n* = 6 (two‐tailed *t*‐test) *p < .05 and **p < .01. (O) Biotin pull‐down assays revealed that Snd1 knockdown attenuates the interaction between circHipk3 and Ptbp1‐3′UTR. BMDMs transfected with either control siRNA or Snd1‐siRNA were incubated with in vitro synthesised biotin‐labelled circHipk3 probe or eGFP probe for the pull‐down assays, followed by real‐time qPCR analysis to examine Ptbp1 mRNA levels. Western blotting of total proteins from cell lysates was conducted using Snd1 antibodies. Data shown are the mean ± SD (*n* = 3; GAPDH as the internal reference). *p < .05, **p < .01, ***p < .001 (Student's *t*‐test). (P) A schematic illustration is presented to depict the proposed model elucidating the regulatory role of circHipk3 in modulating Ptbp1 mRNA stability.

Next, we further elucidate the underlying mechanism by which circHipk3 functions in Macrophages. We performed ChIRP assay and excised the circHipk3‐specific band for mass spectrometry analysis (Figure 7A). After overlapping circHipk3‐specific binding proteins with pyroptosis‐related genes in genecards, we identified 10 overlapping pyroptosis‐related proteins for further analysis (Figure 7B). Subsequently, we conducted further analysis to showcase the differential expression of 10 overlapping pyroptosis‐related proteins in GSE51227 (PMID: 25358394) transcriptome dataset. The PCA revealed a distinct separation of AAA samples and controls into discrete clusters (Figure 7C). Among 10 overlapping pyroptosis‐related proteins, the expression levels of the Stat3 and Tlr2 were different in the GSE51227 dataset (Figure 7D,E). The heatmap and differential representation map depicted provided compelling evidence that Stat3 and Tlr2 exhibit significantly different expression levels in the GSE51227 dataset (Figure 7F,G). qPCR confirmed that Stat3 and Tlr2 level was upregulated in AAA (Figure 7H). We selected Stat3 as a potential target protein due to its upstream role in activating the NLRP3 inflammasome and established involvement in the progression of AAA. According to predictions from the catRAPID website (http://service.tartaglialab.com/page/catrapid_group), circHipk3 may interact with Stat3 (Figure 7I). Furthermore, we then confirmed the interactions between circHipk3 and Stat3 by performing RIP assays, which demonstrated that circHipk3 was enriched with an anti‐Stat3 antibody compared to that achieved with a non‐specific IgG antibody (Figure 7J). Stat3 was found to specifically bind to circHipk3, which was verified by Western blotting (Figure 7K). According to the expression data from the Genotype‐Tissue Expression project (https://gtexportal.org/home/), Stat3 mRNA is highly expressed in the aorta and coronary arteries (Figure 8L). The results of Western bolt indicated that Stat3 was highly expressed in human AAA (Figure 8M). We next investigated whether the interactions between circHipk3 and Stat3 affected Stat3 levels. Enhanced circHipk3 significantly increased Stat3 protein levels in macrophages (Figure 7N). To elucidate the molecular mechanisms underlying the interplay between circHipk3 and Stat3, we subjected circHipk3‐knockdown macrophages to treatment with the protein synthesis inhibitor cycloheximide (CHX). Notably, the half‐life of Stat3 in the circHipk3‐knocked down macrophages was significantly reduced compared to that in the control macrophages, suggesting that circHipk3 exerts a delaying effect on Stat3 degradation. (Figure 7O). Next, we further explored whether overexpression of Stat3 affected the protein expression levels of NLRP3 and IL‐1β, and IL‐18. Our correlation analysis revealed that NLRP3 had the highest level of association with Stat3 among the pyroptosis‐critical genes (Figure 7P,Q). Previous studies have shown that Stat3 was capable of binding to the NLRP3 promoter and enhancing H3K9 acetylation, and NLRP3 transcription, as well as NLRP3/CASP1‐mediated pyroptosis.^19^, ^20^, ^21^, ^22^, ^23^ Overexpression of Stat3 upregulated the protein expression levels of NLRP3, IL‐1β and IL‐18 (Figure 7R–U). Furthermore, to demonstrate that Stat3 promotes the maturation of IL‐1β and IL‐18, we assessed the RNA levels of IL‐1β and IL‐18 in macrophages overexpressing Stat3, and used ELISA to measure the levels of mature IL‐1β and IL‐18 in the cell supernatant. Our findings revealed that overexpression of Stat3 significantly upregulated both the RNA levels in macrophages and the ELISA‐detected levels in the supernatant for IL‐1β and IL‐18 (Supporting Information Figure S11A,B). Overexpression of circHipk3 enhances the expression of NLRP3, IL‐1β and IL‐18; however, this effect is abrogated by the knockdown of Stat3 (Supporting Information Figure S12A). Taken together, the above results suggest that circHipk3 regulates macrophage pyroptosis through the Stat3‐NLRP3‐IL‐1β/IL‐18 pathway.

### 3.7 CircHipk3 facilitates macrophage pyroptosis by promoting Ptbp1 mRNA degradation to inhibit autophagy

Previous studies have demonstrated that intracellular autophagy plays a critical role in clearing excess NLRP3 inflammasome, with its attenuation resulting in the accumulation of NLRP3 inflammasome.^24^, ^25^, ^26^ Additionally, research has shown that circHipk3 inhibits autophagy formation.^27^, ^28^, ^29^ We observed that overexpression of circHipk3 suppresses the expression of autophagy‐related proteins Atg5 and Beclin1 (Figure 8A,B). Conversely, knockdown of circHipk3 promotes autophagy formation, as evidenced by both immunofluorescence analysis (Supporting Information Figure S9B) and increased autophagic flux (Figure 8C). Moreover, we observed with Transmission electron microscope that autophagosomes of macrophages increased significantly after circHipk3 knockdown (Supporting Information Figure S9A), suggesting the inhibition of circHipk3 on autophagy. Using a Venn diagram approach, we identified Ptbp1 and Snd1 as downstream targets of circHipk3 in relation to autophagy regulation (Figure 8D). RIP assays confirmed specific binding of Snd1 and Ptbp1 to circHipk3, as validated by subsequent Western blotting (Figure 8E–G). Bioinformatics predictions, qPCR and Western blot analyses further substantiated elevated levels of Ptbp1 and Snd1 in AAA conditions (Figure 8H–J). Notably, overexpression of circHipk3 was associated with increased Ptbp1 expression, suggesting a positive correlation between circHipk3 levels and Ptbp1 stability. To explore the molecular mechanisms underlying the interaction between circHipk3 and Ptbp1, Previous studies have found that circRNA could influence the stability of Ptbp1 mRNA. we investigated whether circHipk3 could influence the stability of Ptbp1 mRNA and mediate the expression of Ptbp1. Experiments using actinomycin D and dimethylsulfoxide (DMSO) to inhibit RNA synthesis revealed that circHipk3 destroyed Ptbp1 mRNA stability over time (Figure 8K). Further investigation into Snd1's role indicated that it significantly affects Ptbp1 mRNA half‐life in BMDMs (Figure 8L,M). This led us to hypothesise that Snd1 facilitates the interaction between circHipk3 and Ptbp1 mRNA, thereby enhancing Ptbp1 stability. RNA pull‐down assays following Snd1 knockdown in BMDMs confirmed reduced enrichment of Ptbp1 mRNA by the circHipk3 probe, highlighting Snd1's influence on the binding capacity of circHipk3 and Ptbp1‐3′UTR (Figure 8N). Moreover, upon overexpression of circHipk3, we observed a decrease in the levels of the autophagy protein Beclin1. However, this effect was reversed when we Ptbp1 overexpression (Supporting Information Figure S12B). In summary, our findings suggest that Snd1 may modulate Ptbp1 mRNA levels by regulating the interaction between circHipk3 and Ptbp1 RNA molecules.

## 4 DISCUSSION

In this study, we identified circHipk3‐mediated macrophage pyroptosis as the upstream node of inflammatory cytokines and MMP and could promote the synergistic effect of inflammation and MMP synthesis to induce AAA formation and rupture. CircHipk3 augmented macrophage pyroptosis by dual mechanisms: it enhanced NLRP3 inflammasome production by interaction with Stat3 and bound Snd1 to promote Ptbp1 mRNA degradation to inhibit autophagy and subsequently reduced the degradation of NLRP3 inflammasome. Therefore, circHipk3 was implicated in both the source and degradation of NLRP3, making it a more potential therapeutic target for pyroptosis intervention and preventing AAA formation.

We verified circRNA (circHipk3) involved in regulating macrophage pyroptosis in macrophages and AAA models. In this study, we found that circHipk3 downregulation significantly suppressed pyroptosis markers (NLRP3, GSDMD and cleaved CASP1) in vitro of macrophage and in Ang II‐ and PPE‐induced AAA model, whereas upregulated of circHipk3 increased NLRP3, GSDMD and cleaved CASP1 expression level in Ang II‐ and PPE‐induced AAA model, which suggested that circHipk3 might contribute to the activation of macrophage pyroptosis in AAA. In human aneurysm specimens, we also found that both pyroptosis markers (NLRP3, GSDMD and CASP1) and circHipk3 were mainly concentrated in the adventitia of aneurysm tissue, which was the main site of macrophage infiltration. These results showed that the distribution of macrophage pyroptosis and circHipk3 is highly consistent, which provided the possibility for circHipk3‐mediated macrophage pyroptosis in AAA. Previous studies showed that pyroptosis is a pro‐inflammatory pathological process, which showed inflammatory pathological changes (such as necrosis) in morphology.^30^ Our results showed that the knockdown of circHipk3 significantly decreased the macrophage necrosis rate and the trend of macrophage necrosis and pyroptosis after circHipk3 downregulation are consistent. These results partially supported that circHipk3 might promote macrophage pyroptosis in AAA. The previous study also demonstrated that circHipk3 accelerates the production of CASP1 and NLRP3 in macrophages in gouty arthritis. Besides, circHipk3 could interact with several pyroptosis miRNAs such as miR‐124,^15^ miR‐193a,^16^ and miR‐192,^17^ which further support the potential of circHipk3 to promote pyroptosis. Thus, these results demonstrated the ability of circHipk3 to promote macrophage pyroptosis in AAA. Additionally, it is noteworthy that our previous studies have demonstrated that circular RNA Cdyl mediates the formation of AAA by regulating macrophage polarisation. This further suggests that circRNA may play a pivotal role in AAAs. Moreover, macrophage polarisation and macrophage pyroptosis are two critical pathological processes in the inflammatory response of macrophages. There are some shared key molecular proteins in these processes, which further supports the conclusion that circRNA exerts its function in AAA through pyroptosis.

We identified that macrophage pyroptosis could promote the synergistic effect of inflammation and MMP synthesis to induce AAA formation. In our study, circHipk3 overexpression upregulated pyroptosis marker GSDMD, CASP1, inflammatory cytokines IL‐1β, IL‐18, MMP2/9 and macrophage marker CD68 in vitro of macrophage and AAA model, promoted elastin fibres degradation, increased the abdominal aorta average maximum diameter and the incidence rate of the aneurysm in PPE‐ and Ang II‐induced AAA models. Whereas genetic downregulation of circHipk3 displayed the opposite effect in vivo. These results suggested that circHipk3 could aggravate inflammation and MMP by augmenting macrophage pyroptosis to promote AAA formation. Previous studies have shown that interference of circHipk3 suppresses inflammation by inhibiting pyroptosis in septic acute kidney injury^15^ and gouty arthritis.^17^ These studies support our discovery to some extent. Importantly, we further found that macrophage pyroptosis promoted the synergistic effect on inflammation and MMP in AAA. Our results showed inflammatory cytokines and MMP2/9 were synchronously suppressed after macrophage pyroptosis silence in vitro and circHipk3 deficiency attenuate synergistic effect on cytokines and MMP synthesis by suppressing macrophage pyroptosis to degrade elastic fibres. Previous studies have shown that inhibiting the pyroptosis suppressed synergistic effect of inflammatory cytokines and MMP2/9 to attenuate cardiac dysfunction in pressure overload,^31^ which partially supported our finding. Therefore, inhibiting macrophage pyroptosis might attenuate the synergistic effect of inflammation and MMP synthesis to suppress AAA formation.

Intriguing, we found that macrophage pyroptosis promoted inflammation and MMP to participate in the entire pathological process of AAA. Our results showed that circHipk3 overexpression upregulated pyroptosis markers, inflammatory cytokines, MMP2/9 and macrophage content in the AAA model. A previous study found NLRP3 inflammasome activation and macrophage pyroptosis trigger inflammatory cytokines IL‐1β, and IL‐18 secretion, and initiate the formation of AAA.^6^ Subsequently, the synergistic effects of macrophage pyroptosis and inflammatory cytokines IL‐1β and IL‐18 promoted inflammatory cell synthesis MMP2/9, which further contributes to the degradation of elastin fibres, exacerbating the progression and enlargement of the AAA.^32^, ^33^ Thus, these results suggested circHipk3‐mediated macrophage pyroptosis might be involved in early and mid‐stage lesions of AAA disease. Moreover, we also found upregulation of circHipk3 increased the rupture rate of aortic aneurysms and promoted abdominal aortic dissection formation in Ang II‐induced AAA models. Theoretically, as the lesion progresses in the late stages, inflammatory cytokines and MMP2/9 induced smooth muscle cells apoptosis, collagens fibre degradation and neovascularisation, which are key factors in the late‐stage development of aneurysms, such as aneurysm rupture and aortic dissection.^34^, ^35^ These results further provide evidence to support circHipk3 might inhibit the late‐stage progression of AAA disease. Collectively, circHipk3 reduced the inflammation and MMP by intervening macrophage pyroptosis to prevent initiation and progression of AAA and might be a more potential therapeutic target.

Mechanically, the present study revealed that circHipk3 enhanced NLRP3 inflammasome production by interaction with Stat3 and bound Snd1 to promote Ptbp1 mRNA degradation to inhibit autophagy and subsequently reduced the degradation of NLRP3 inflammasome. Using the dual validation of CHIRP and RIP assays, mass spectrometry analysis and bioinformatics, we innovatively discovered that Stat3 is a downstream target protein in circHipk3. Furthermore, overexpression of circHipk3 significantly increased Stat3 protein levels. These results suggested a positive correlation between the expression levels of circHipk3 and Stat3. Previous studies have found that circHipk3 could enhance Stat3 expression level by regulating the miR‐124^29^, ^36^ and miR‐637,^37^, ^38^ which partially supported the positively correlated relationship between circHipk3 and Stat3. Our results suggested that circHipk3 exerts a delaying effect on Stat3 degradation, which is supported by the molecular mechanisms underlying the interplay between circFOXP1 and Stat3.^39^ These results indicated that circRNA mainly expressed in the cytoplasm could bind to Stat3 to inhibit Stat3 protein degradation, enhance the stability of Stat3 protein and promote the expression of Stat3 protein. We applied Western blotting, qPCR and microarray technology to detect the difference expression level of Stat3 in the AAA tissue and control group. Stat3 was reported to be capable of binding to the NLRP3 promoter and enhanced NLRP3 transcription to participate in neuronal pyroptosis,^19^ mechanical allodynia,^40^ and Parkinson's disease.^41^ These results helped to confirm the pro‐pyroptosis potential effects of Stat3 on AAA formation and avoid false positive results. Additionally, our investigation revealed that circHipk3 exerts inhibitory effects on autophagy, thereby diminishing NLRP3 degradation through its interaction with Snd1, which in turn facilitates Ptbp1 mRNA degradation, thus fostering pyroptosis. Previous research has underscored the significance of both circHipk3^27^, ^28^ and Ptbp1^42^, ^43^ in the realm of autophagy, further corroborating our findings. Moreover, existing literature has documented the regulatory role of circRNA in Ptbp1 mRNA degradation,^44^ lending support to our study's assertions. Collectively, the role of circHipk3 in macrophage pyroptosis is multifaceted, as it interacts with Stat3 to enhance pyroptotic cell death, elevates NLRP3 levels and simultaneously binds to Snd1, facilitating the degradation of Ptbp1 mRNA and thereby inhibiting autophagy. Previous research has established that the balance between the generation and degradation of inflammasomes is pivotal in determining the intracellular levels of these complexes. Modulating either process in isolation may not be sufficient to significantly reduce inflammasome content. Autophagy, in particular, can negatively regulate inflammasome activation by degrading the components of the inflammasome complex, thus serving as a check on excessive inflammation. Given these insights, the simultaneous regulation of inflammasome generation and autophagy emerges as a potentially effective strategy for reducing intracellular NLRP3 levels. Furthermore, circHipk3, with its identified roles in both NLRP3 production and autophagy inhibition, may serve as a key regulatory factor capable of modulating these dual pathways to reduce inflammasome content. CircHipk3's multifunctional effects on macrophage pyroptosis and inflammasome regulation underscore the complexity of cellular responses to inflammatory stimuli. Its ability to influence both the production of NLRP3 and the autophagic process positions it as a potential therapeutic target for modulating inflammatory responses and preventing excessive inflammation‐associated pathologies. Further investigation into the mechanisms by which circHipk3 exerts these effects is warranted to fully harness its potential in the context of inflammatory and immune‐related disorders. In summary, circHipk3 was implicated in both the source and degradation of NLRP3, making it a promising target for pyroptosis intervention.

Nevertheless, there are still some limitations in our study. First, the elucidation of upstream signalling pathways and a more specific mechanism of the regulation of circHipk3 expression in AAA formation may require further investigation. Second, molecular sponge mechanism was also verified to be an important way to mediate circRNA action and numerous of miRNAs had been confirmed to be key contributors for the aneurysm pathology. Although we have suggested that circHipk3 played its regulatory role in macrophage pyroptosis by the interaction with downstream protein, it was still a meaningful topic to look into the possibility of circHipk3 acting on miRNAs.

In conclusion, our findings suggest that circHipk3 plays a pivotal role in promoting the synergistic effect of cytokine secretion and MMP synthesis, thereby exacerbating macrophage pyroptosis and contributing to AAA formation. Mechanistically, circHipk3 enhances this synergistic effect by interaction with Stat3, increase the NLRP3 level in the aorta, and by binding Snd1 to promote Ptbp1 mRNA degradation to inhibit autophagy, consequently fostering macrophage pyroptosis. Given its central involvement in AAA pathogenesis, circHipk3 emerges as a promising therapeutic target for mitigating AAA formation and rupture. Efforts aimed at modulating circHipk3 levels or activity hold potential for preventing and managing this debilitating vascular condition.

## AUTHOR CONTRIBUTIONS

Jianping Bin and Xiang He: manuscript writing; Donghua Cai, Jianping Bin and Xiang He: conception and design; Chuling Li, Sisi He and Yihai Guo: experimental design and data acquisition; Wangjun Liao, Yulin Liao and Jianping Bin: supervision. All the authors take full responsibility for the work.

## CONFLICT OF INTEREST STATEMENT

The authors declare no conflicts of interest.

Data will be made available upon request.

## ETHICS STATEMENT

Human aortic tissue samples were obtained in the context of a multicentre clinical study, which received ethical clearance from the Ethics Committees of Nanfang Hospital, with the assigned approval number NFEC‐2019‐086. Furthermore, all animal‐related procedures were carried out with the consent of the Institutional Animal Care and Use Committee at Southern Medical University, ensuring compliance with ethical standards for animal research.

## Supporting information



Supporting Information

## Data Availability

The public RNA‐seq and single‐cell RNA‐seq data in this study have been appropriately archived in the NCBI GEO database. The accession codes for these datasets are GSE51227 and GSE152583, respectively. These repositories provide a valuable resource for the research community to access and verify the results of your study. For those interested in accessing the data, here are the direct links to the datasets: GSE51227: (https://www.ncbi.nlm.nih.gov/geo/query/acc.cgi?acc=GSE51227) GSE152583: (https://www.ncbi.nlm.nih.gov/geo/query/acc.cgi?acc=GSE152583). Additionally, the statement that all other data supporting the key findings are available within the article and supplemental information files, or from the corresponding author upon reasonable request, ensures transparency and reproducibility of the study's results. The availability of data points in graphs reported in the Supporting Information file further facilitates the scrutiny and reuse of the study's data by other researchers. This practice aligns with the principles of open science and contributes to the integrity of the scientific record.
