# Supplementary material for: CircHipk3 serves a dual role in macrophage pyroptosis by promoting NLRP3 transcription and inhibition of autophagy to induce abdominal aortic aneurysm formation
Source: Clin Transl Med. 2024 Nov 27;14(12):e70102. doi: 10.1002/ctm2.70102 (PMC11599875; doi:10.1002/ctm2.70102)
Supplement: Supplementary file 1 — Supporting Information [file CTM2-14-e70102-s001.docx]

Supplemental Information

CircHipk3 serves a dual role in macrophage pyroptosis by promoting NLRP3 transcription and inhibition of autophagy to induce abdominal aortic aneurysm formation

Donghua Cai, Chuling Li Yingyuan Zhang, Sisi He, Yihai Guo, Wangjun Liao, Yulin Liao, Jianping Bin, Xiang He

| **Table I. Patient clinical information（n=10）** | | | | | | | | |
| --- | --- | --- | --- | --- | --- | --- | --- | --- |
| patient | gender | age | smoking status | aortic  Diameter  (mm) | Hyper  lipidemia | Hyper  tensive | coronary artery disease |  |
| ID1 | female | 72 | no | 60 | no | no | no |  |
| ID2 | male | 69 | yes | 62 | yes | yes | no |  |
| ID3 | male | 71 | yes | 61 | yes | yes | no |  |
| ID4 | male | 61 | no | 57 | yes | no | no |  |
| ID5 | male | 60 | yes | 68 | yes | yes | yes |  |
| ID6 | male | 52 | no | 60 | no | no | no |  |
| ID7 | male | 63 | no | 56 | no | no | no |  |
| ID8 | male | 70 | yes | 72 | yes | yes | yes |  |
| ID9 | male | 68 | yes | 69 | yes | yes | yes |  |
| ID10 | male | 68 | yes | 70 | yes | yes | yes |  |

**Table II. Quantitative real-time PCR and Specific siRNAs**

|  | Forward | Reverse |
| --- | --- | --- |
| circHipk3  (mouse) | CAGCACCGTAACCATACTT | TGGTGGGTAGACCAAGACTTGTGA |
| circHipk3  (human) | CAGCACCGTAACCATACTT | TGGTGGGTAGACCAAGACTTGTGA |
| NLRP3  (human) | GAT CTT CGC TGC GAT CAA CAG | CGT GCA TTA TCT GAA CCC CAC |
| Caspase-1  (human) | TCC AAT AAT GGA CAA GTC AAG CC | GCT GTA CCC CAG ATT TTG TAG CA |
| Caspase-1  (mouse) | ACA AGG CAC GGG ACC TAT G | TCC CAG TCA GTC CTG GAA ATG |
| IL-1β  (human) | CCA AAG AAG AAG ATG GAA AAG C | GGT GCT GAT GTA CCA GTT GGG |
| IL-1β  (mouse) | CAACCAACAAGTGATATTCTCCATG | GATCCACACTCTCCAGCTGCA |
| IL-18  (mouse) | GGAGTCCACTGGCGTCTTCA | GTCATGAGTCCTTCCACGATACC |
| Ptbp1  (mouse) | AGAGGAGGCTGCCAACACTA | ATTGCCATTCCTGCATCCA |
| Snd1  (mouse) | TGTGCCACTGTCACCATTGGAG | CAGCTCATCGTAGTGTGAAGACC |
| GADPH  (human) | GGAGTCCACTGGCGTCTTCA | GTCATGAGTCCTTCCACGATACC |
| ShcircHipk3/si-circHipk3 | Sequence(5’-3’) TACTACAGGTATGGCCTCA | |
| circHipk3（mouse） | mmu_circ_0001052 | |
| circHipk3（human） | hsa_circ_000284 | |

| **Table III. Antibodies for western blots** | | |
| --- | --- | --- |
| name | Vendor or Source | Catalog # |
| anti-NLRP3  (human and mouse) | Abcam | ab263899 |
| anti-caspase-1  (mouse) | Proteintech | 22915-1 AP |
| anti-MMP9 (human and mouse) | Bios | bs-4593R |
| anti-MMP2 (human and mouse) | Bios | bs-0412R |
| anti-GSDMD (mouse) | Abcam | ab219800 |
| anti-IL-1β (mouse) | Bios | bs-0812R |
| anti IL-18 (mouse) | Beyotime | AF7266 |
| anti Stat3 (mouse) | Abcam | ab68153 |
| anti Atg5 (mouse) | Abcam | ab108327 |
| anti Beclin1 (mouse) | Abcam | ab207612 |
| anti Ptbp1 (mouse) | Abcam | ab133734 |
| anti Snd1 (mouse) | Abcam | ab225620 |
| anti-GAPDH（human and mouse） | Bios | bs-2188R |

| **Table IV. Antibodies for immunofluorescent analysis** | | |
| --- | --- | --- |
| name | Vendor or Source | Catalog # |
| Anti-NLRP3 (mouse) | Bios | bs-10021R |
| Anti- caspase-1(human and mouse) | Proteintech | 22915-1 AP |
| Anti-cd68 (mouse) | Bio-Rad | MCA1957GA |

**Table V. Antibodies for immunohistochemistry analysis**

| name | Vendor or Source | Catalog # |
| --- | --- | --- |
| Anti-NLRP3 (human and mouse) | Bios | bs-10021R |
| Anti- caspase-1(human and mouse) | Proteintech | 22915-1 AP |
| Anti-GSDMD (human and mouse) | Abcam | ab219800 |
| Anti-IL-1β (human and mouse) | Bios | bs-0812R |
| Anti-MMP9 (mouse) | Bios | bs-4593R |
| Anti-MMP2 (mouse) | Bios | bs-0412R |
| Anti-cd68 (mouse) | Bio-Rad | MCA1957GA |

Supplemental figures and legends


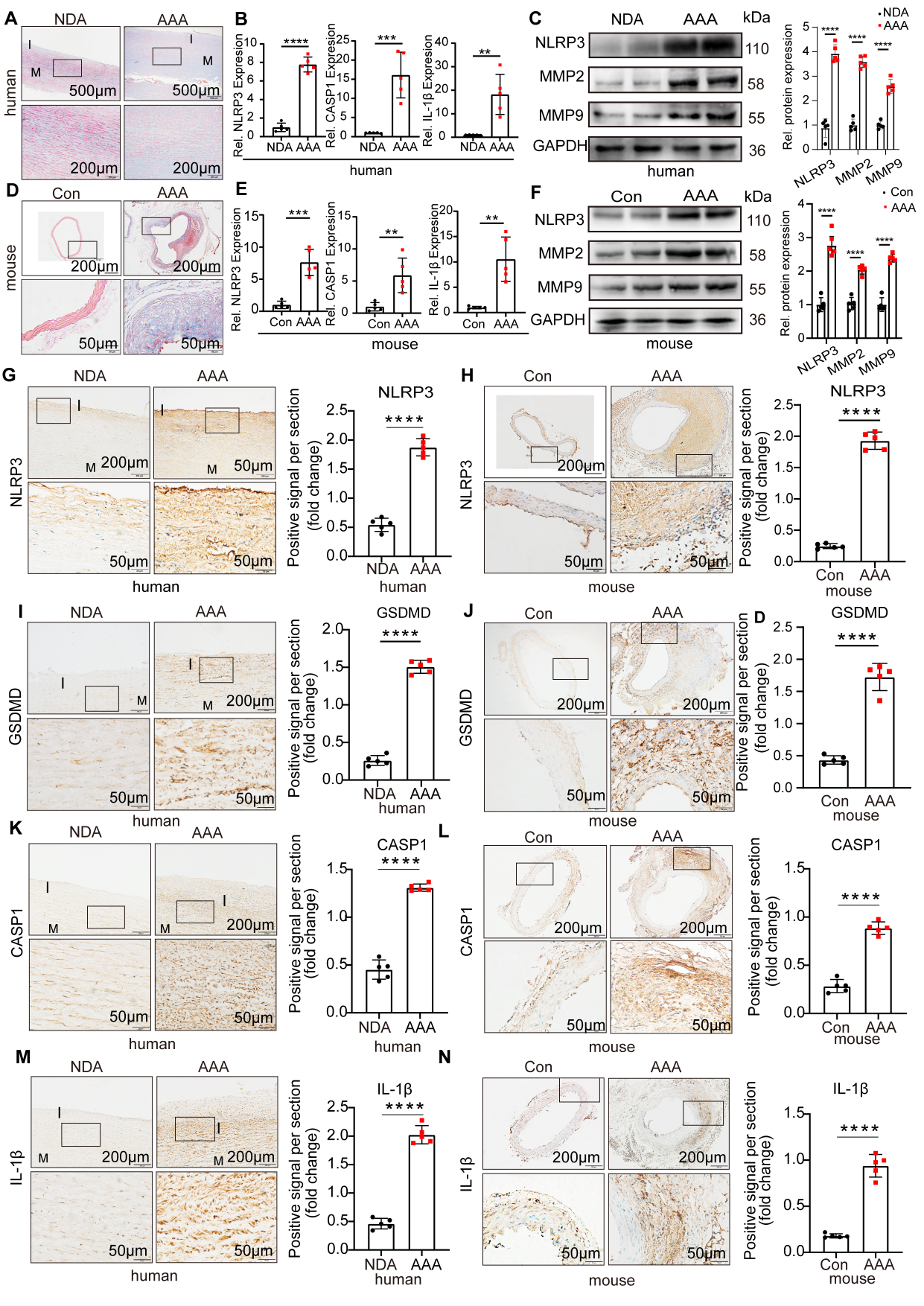


Supplemental Figure 1. Elevated expression of pyroptosis markers and increased fibrosis are observed in both human abdominal aortic aneurysm and ang-II-induced mouse AAA.

(A) Representative staining with Masson’s Trichrome (collagen) in the human AAA tissues or tissues. (B) Relative expression of pyroptosis makers NLRP3, CASP1and IL-1β in human AAA and NDA tissues (qPCR). ***p*<0.01, ****p*<0.001, *****p*<0.0001 versus the NDA group,n=5 per group. (C) Western blot and densitometric analysis of NLRP3, MMP-2, MMP-9 in human NDA and AAA tisues. (GAPDH as the internal reference)*****p*<0.0001, n= 5 per group. (D) Representative staining with Masson’s Trichrome (collagen) in the suprarenal aortas of mice after saline or Ang II infusion. (E) Relative expression of pyroptosis makers NLRP3, CASP1and IL-1β in mouse AAA and NA tissues (qPCR). ***p*<0.01, ****p*<0.001 versus the NA group ,n=5 per group. (F) Western blot and densitometric analysis of NLRP3, MMP-2, MMP-9 in mouse NA and AAA tisues. (GAPDH as the internal reference) .*****p*<0.0001,n=5 per group. (G,I,K,M) Immunohistochemistry staining for NLRP3, GSDMD, CASP1, IL-1β in normal human aortas and human AAA aortas (bars: upper 200 μm, lower 50 μm). ***p*<0.01,****  *p* <0.0001 .(H,J,L,N) Immunohistochemistry staining for NLRP3, CASP1, IL-1β in normal ApoE-/- mouse aortas and ApoE-/- mouse aortas with Ang II stimulation. (bars: upper 200 μm, lower 50 μm, ) . ** *p*<0.01,*****p*<0.0001.


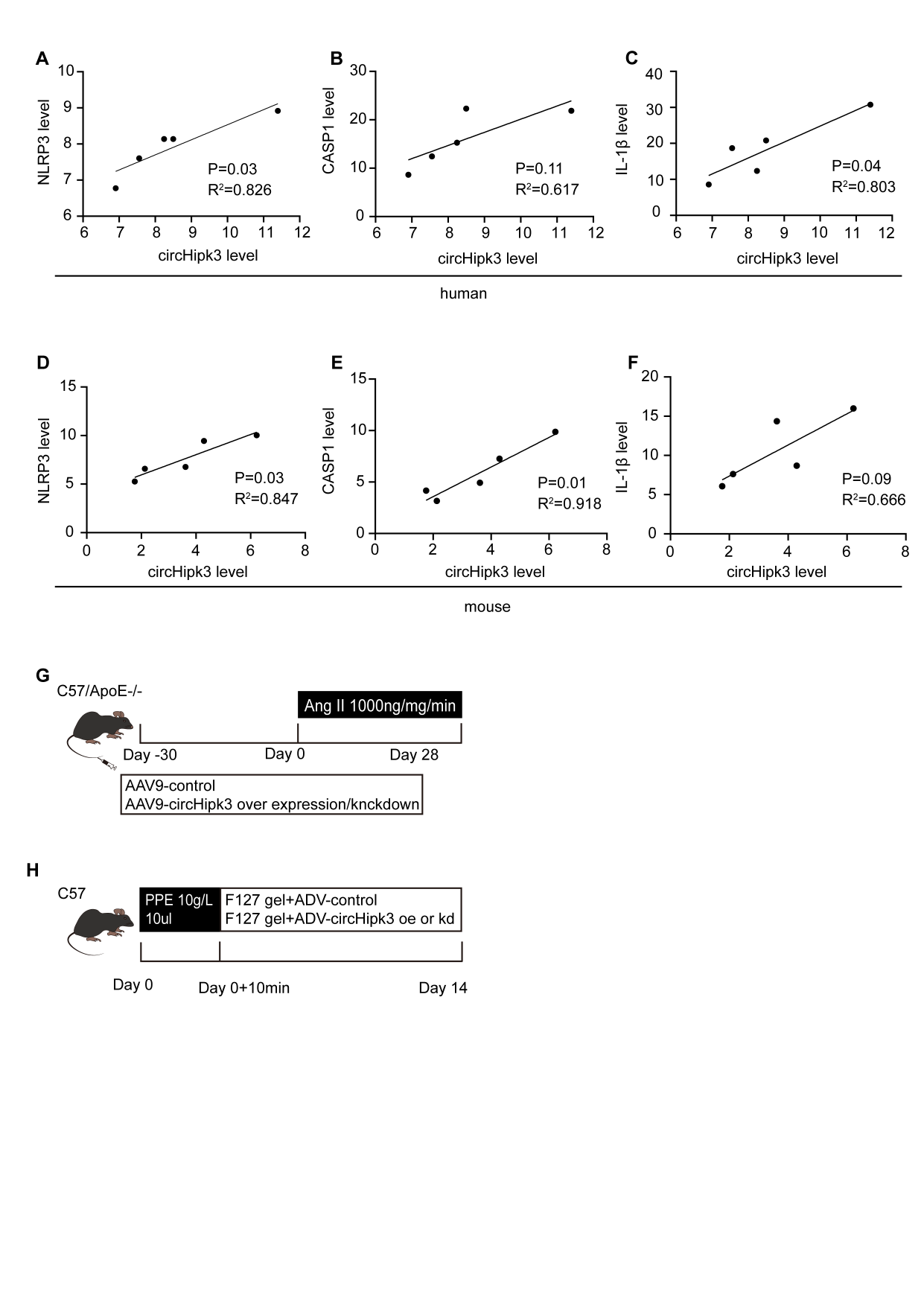


Supplemental Figure 2. The expression levels of circHipk3 in human and mouse samples are correlated with the pyroptosis markers NLRP3, CASP1, and IL-1β.

(A-C) The correlation between circHipk3 and NLRP3,circHipk3 and Caspase-1,circHipk3 and IL-1β expression in human AAA tissue. (D-F) The correlation between circHipk3 and NLRP3,circHipk3 and CASP1,circHipk3 and IL-1β expression in mice AAA tissue. (G) Thirty days prior to the establishment of the AngII-infused mouse AAA model, Adeno-associated virus 9 carrying the circHipk3 knockdown/overexpression construct was administrated intravenously into ApoE- /- and C57BL/6 J mice using an insulin syringe and a 30-gauge needle.(H) The adventitia of the isolated abdominal aortae was treated with ten microlitres of PPE stock solution for a duration of 10 minutes. This was followed by the perivascular application of the circHipk3 knockdown/overexpression adenovirus, which was dissolved in 30% Pluronic gel solution, to the infrarenal aortas lasting for 14 days.


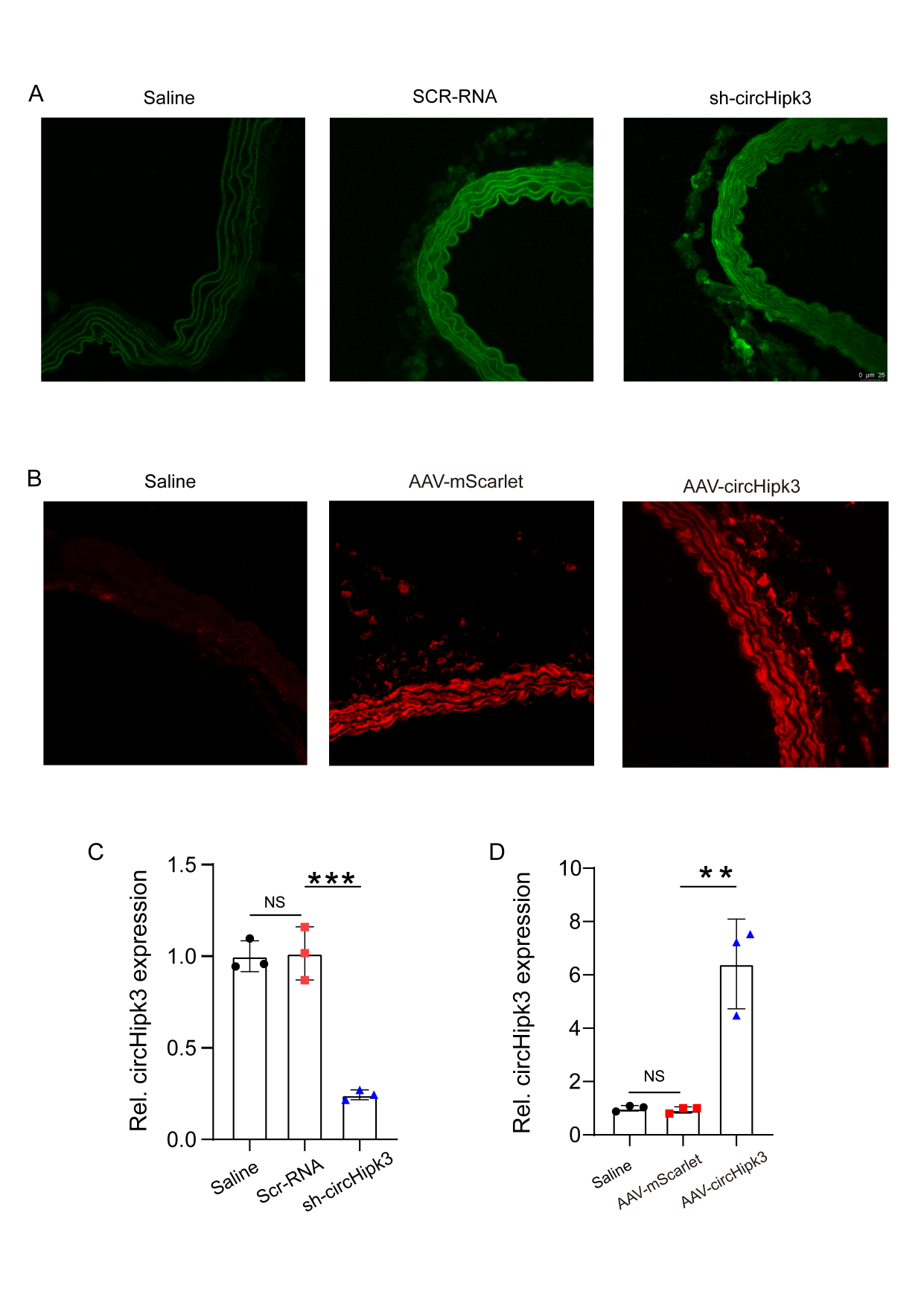


Supplemental Figure 3. The transfection status of AAV (Adeno-Associated Virus) vectors for overexpression and knockdown of circHipk3 in the abdominal aorta of mice.

(A, B) Immunofluorescence staining of virus-borne green fluorescentprotein (GFP) or red flurescentprotein(mScarlet) in the suprarenal aortas of male mice in different Adeno-associated virus-mediated groups and the saline group. (C) The expression of circHipk3 in aortas when circHipk3 was knocked down.,NS,no significant, NS, not significant,****p* < 0.001 vs control group; n=3 per group (one-way ANOVA). (D) The expression of circHipk3 in aortas when circHipk3 was overexpressed. NS, not significant,** *p*< 0.01 vs control group; n=3 per group (one-way ANOVA).


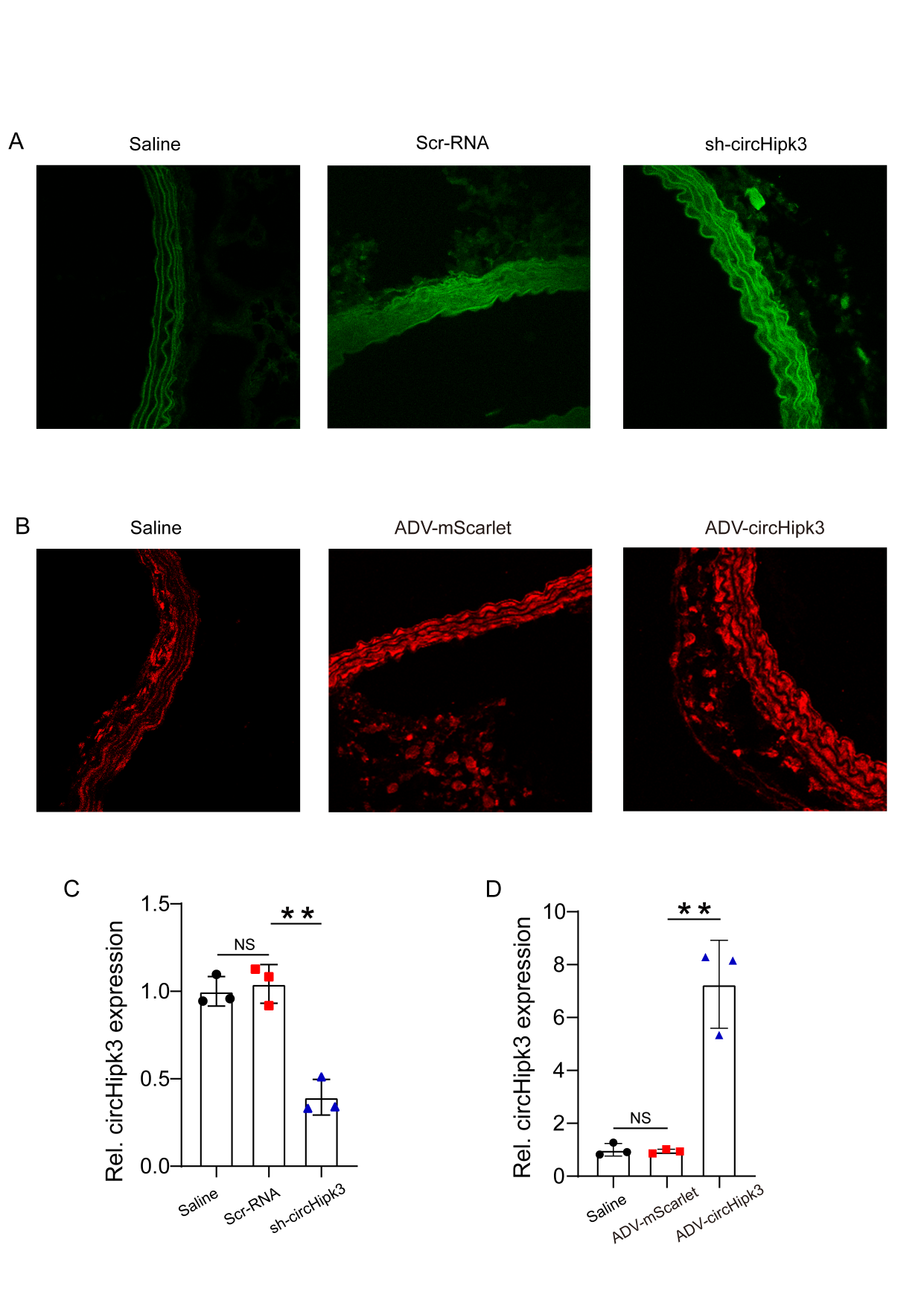


Supplemental Figure 4. The transfection status of ADV (Adenoviral Vectors) for overexpression and knockdown of circHipk3 in the abdominal aorta of mice.

(A, B) Immunofluorescence staining of virus-borne green fluorescentprotein (GFP) or red flurescentprotein (mScarlet) in the suprarenal aortas of male mice in different adenovirus-mediated groups and the saline group. (C) The expression of circHipk3 in aortas when circHipk3 was knocked down. NS, not significant,** *p* < 0.01 vs control group; n=3 per group (one-way ANOVA). (D) The expression of circHipk3 in aortas when circHipk3 was overexpressed. NS, not significant, ***p* < 0.01 vs control group; n=3 per group (one-way ANOVA).


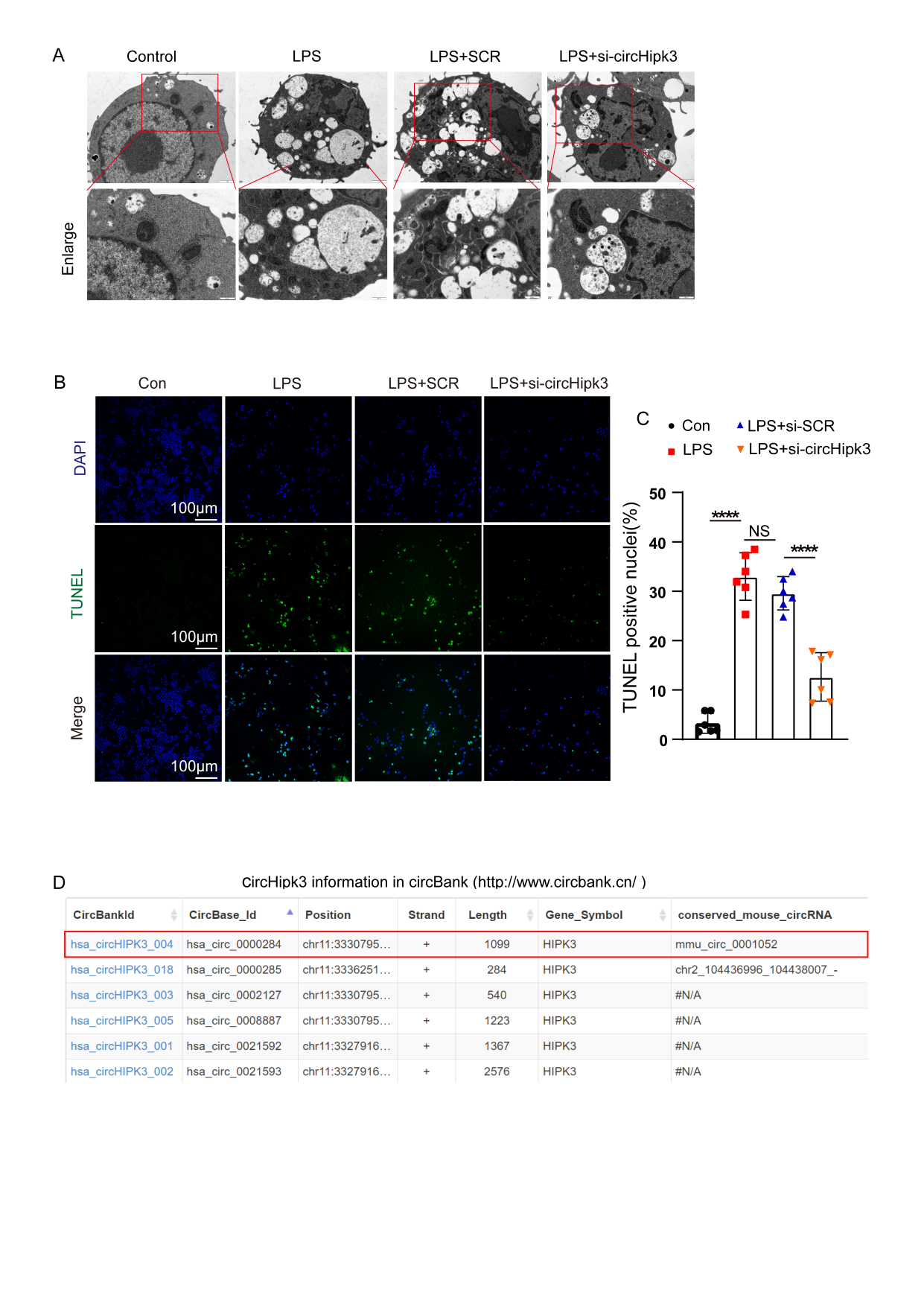
Supplemental Figure 5. Observation of macrophage pyroptosis by transmission electron microscopy, detection of macrophage apoptosis by TUNEL staining, and presentation of circHipk3 information.

BMDMs were transfected with either si-SCR or circHipk3 small interfering RNA fragments for 24 hours, followed by treatment with lipopolysaccharide (LPS) at a concentration of 500 ng/ml for an additional 24 hours.(A) Representative transmission electron microscopy (TEM) images from each group of BMDMs, illustrating the characteristic features of pyroptotic cells.. (B-C) TUNEL staining for macrophage in control group, LPS group,LPS+SCR group and LPS+si-circHipk3 group (bar=100µm) . NS, not significant, *****p* < 0.0001;n=6 per group. (D) circHipk3 information referenced in circBank( http://www.circbank.cn/)


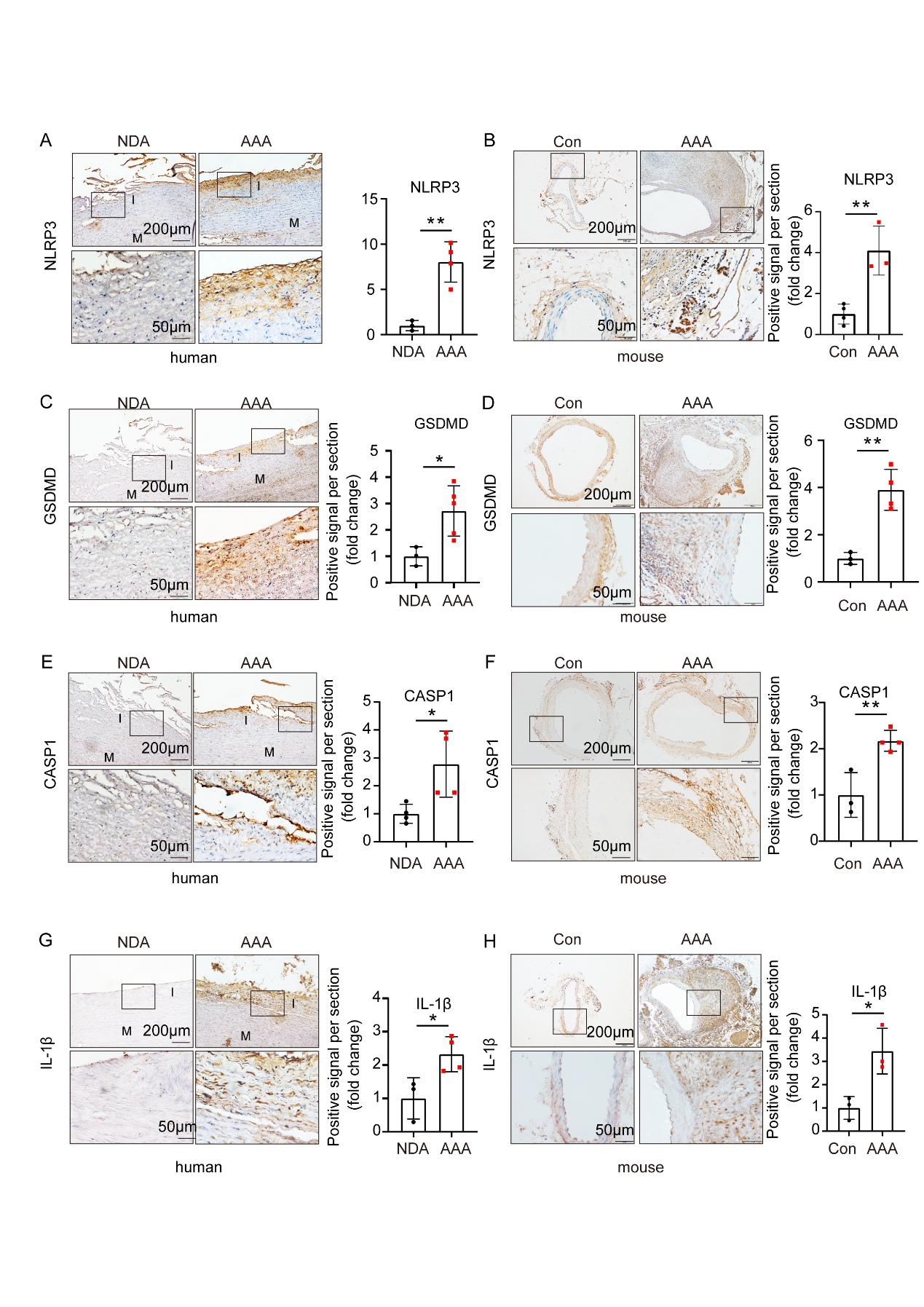


Supplemental Figure 6. Expand the sample size to conduct immunohistochemical staining for pyroptosis markers in both human and murine samples.

(A,C,E,G) Immunohistochemistry staining for NLRP3, GSDMD,CASP1, IL-1β in normal human NDA tissues and human AAA tissues (bars: upper 200 μm, lower 50 μm, n=3 NDA group,n=4 AAA group). **p*<0.05 .(H,J,L,N) Immunohistochemistry staining for NLRP3, CASP1, IL-1β in normal ApoE-/- mouse aortas and ApoE-/- mouse aortas with Ang II stimulation. (bars: upper 200 μm, lower 50 μm, n=3 Con group,n=4 AAA group) . **p*<0.05,** *p*<0.01.


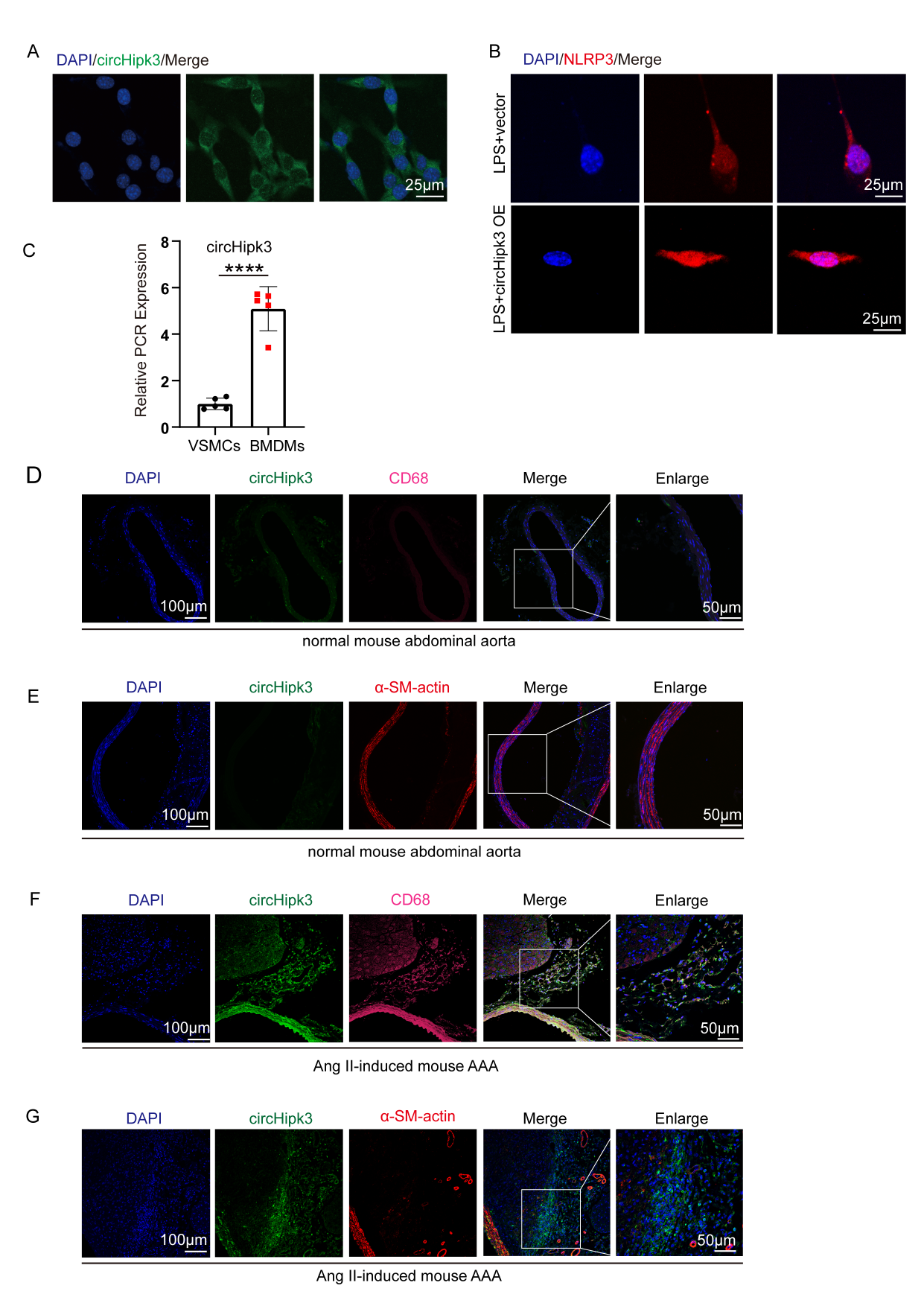


Supplemental Figure 7. circHipk3 is primarily expressed in the cytoplasm of VSMCs with lower levels compared to macrophages, and overexpression of circHipk3 promotes NLRP3 production in VSMCs. circHipk3 is lowly expressed in normal mouse abdominal aortas, highly expressed in macrophages in Ang-II-induced AAA, and lowly expressed in smooth muscle cells.

**(A**) Fluorescence in situ hybridization to determine the circHipk3 levels in macrophages. (bar=25µm) .(B) Immunofluorescence results for NLRP3 in  Vascular Smooth Muscle Cells (VSMCs) transfected with either the vector control or the circHipk3 overexpression plasmid, followed by stimulation with lipopolysaccharide (LPS) at a concentration of 500 ng/mL for 24 hours post-transfection. (bar=25µm). (C) The relative expression of circHipk3 in BMDMs and VSMCs (qPCR). *****p* < 0.0001, n=5 per group.(D) Fluorescence in situ hybridization and Immunofluorescence results of circHipk3, and CD68 in normal mouse abdominal tissues (bars: left 100 μm, right 50 μm, magnified image). (E) Fluorescence in situ hybridization and Immunofluorescence results of circHipk3 and α-SM-actin in normal mouse abdominal tissues (bars: left 100 μm, right 50 μm, magnified image). (F) Fluorescence in situ hybridization and Immunofluorescence results of circHipk3 and CD68 in And II induced mouse AAA tissues (bars: left 100 μm, right 50 μm, magnified image). (G) Fluorescence in situ hybridization and Immunofluorescence results of circHipk3 and α-SM-actin in And II induced mouse AAA tissues (bars: left 100 μm, right 50 μm, magnified image).


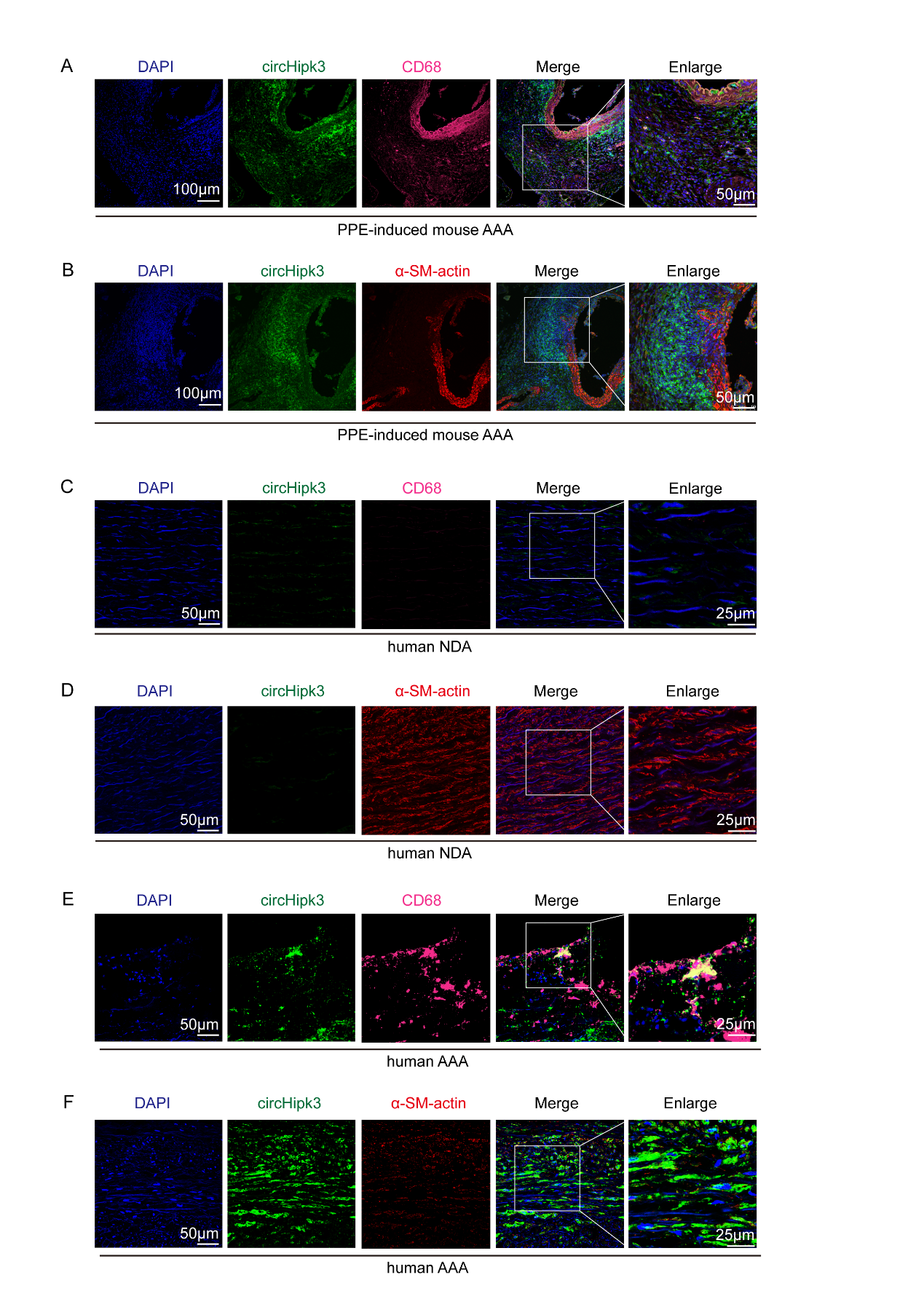


Supplemental Figure 8. In mouse PPE-induced AAA and human NDA/AAA samples, circHipk3 is lowly expressed in normal tissues, highly expressed in macrophages in AAA, and lowly expressed in smooth muscle cells.(A) Fluorescence in situ hybridization and Immunofluorescence results of circHipk3, and CD68 in PPE-induced mouse AAA tissues (bars: left 100 μm, right 50 μm, magnified image). (B) Fluorescence in situ hybridization and Immunofluorescence results of circHipk3 and α-SM-actin in PPE-induced mouse AAA tissues (bars: left 100 μm, right 50 μm, magnified image). (C) Fluorescence in situ hybridization and Immunofluorescence results of circHipk3 and CD68 in And human NDA tissues (bars: left 50 μm, right 25 μm, magnified image). (D) Fluorescence in situ hybridization and Immunofluorescence results of circHipk3 and α-SM-actin in A human NDA tissues (bars: left 50 μm, right 25 μm, magnified image). (E) Fluorescence in situ hybridization and Immunofluorescence results of circHipk3 and CD68 in human AAA tissues (bars: left 50 μm, right 25 μm, magnified image). (F) Fluorescence in situ hybridization and Immunofluorescence results of circHipk3 and α-SM-actin in in human AAA tissues (bars: left 50 μm, right 25 μm, magnified image).


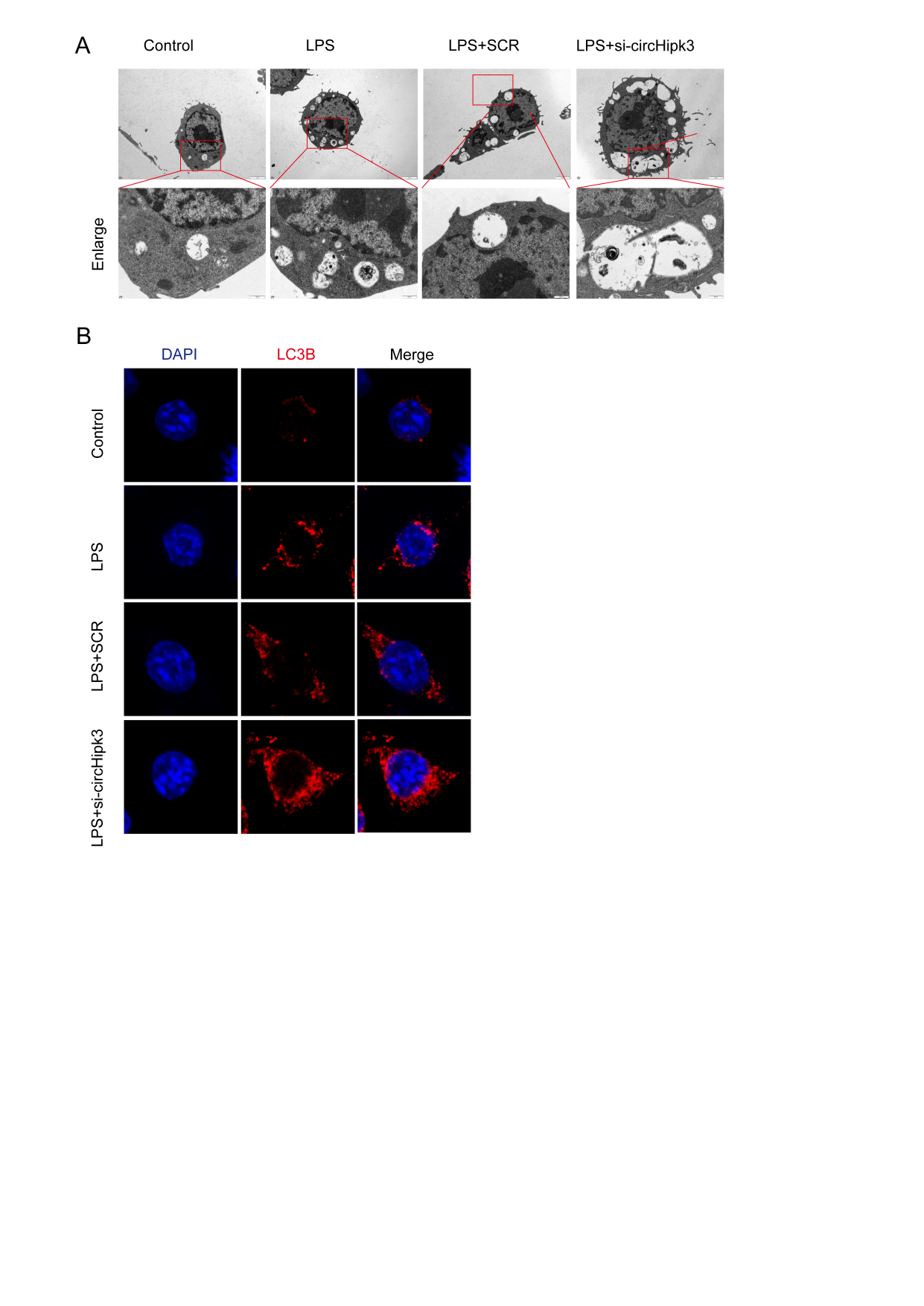


Supplemental Figure 9. Knockdown of circHipk3 promotes the formation and increase of autophagosomes in macrophages.

BMDMs were transfected with either si-SCR or circHipk3 small interfering RNA fragments for 24 hours, followed by treatment with LPS at a concentration of 500 ng/ml for an additional 24 hours.

(A) Representative transmission electron microscopy (TEM) images displaying autophagic features in BMDMs from each group.(B) Immunofluorescence results for LC3B in BMDMs from each group.


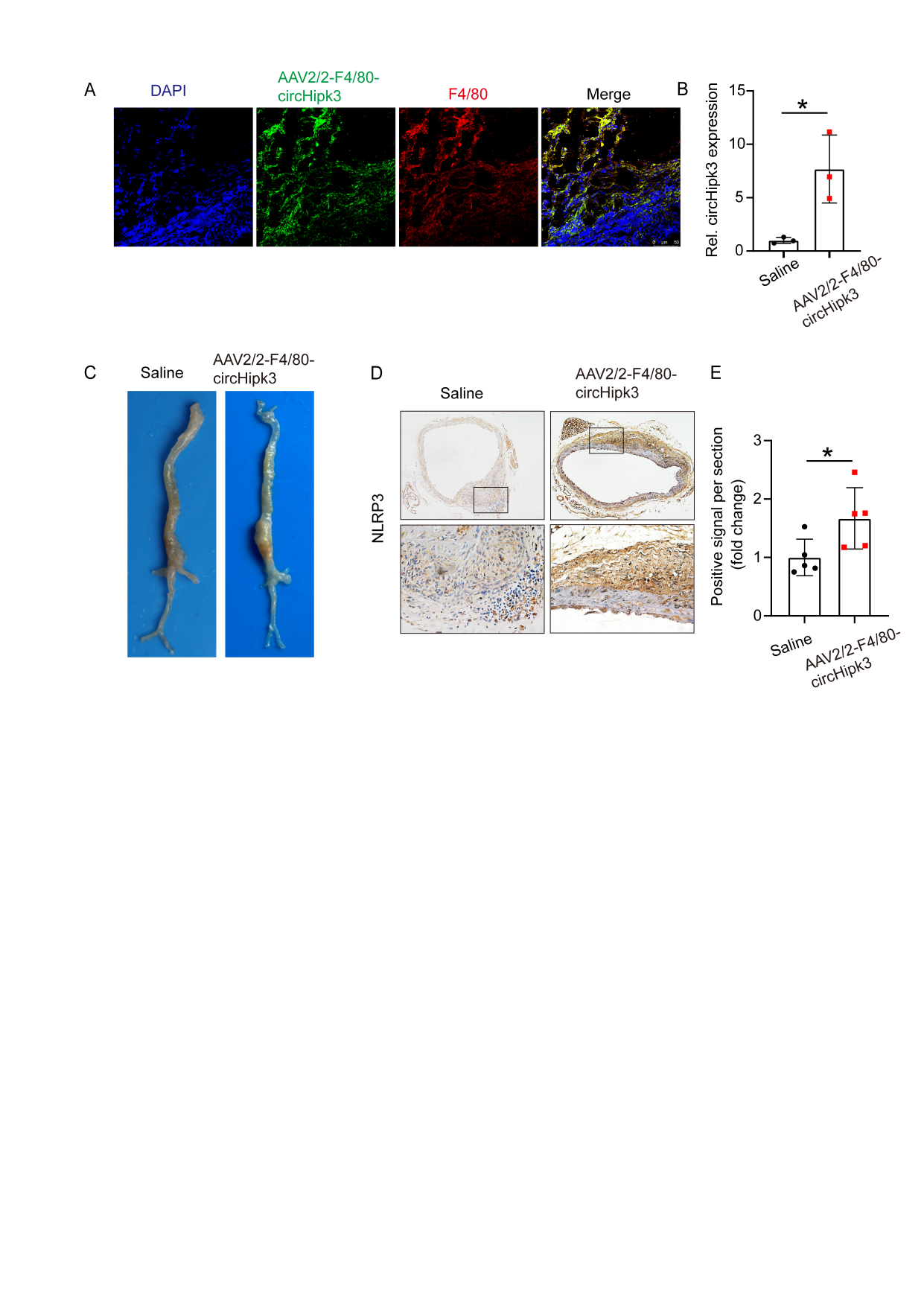


Supplemental Figure 10. The overexpression of circHipk3 carrying a macrophage-specific promoter promotes Ang II-induced AAA formation and increases the levels of NLRP3.

The HBAAV2/2-F4/80 virus, designed with a macrophage-specific promoter based on the circHipk3 sequence .(A) Immunofluorescence staining of virus-borne green fluorescentprotein (GFP) and f4/80 (red). (B)The expression of circHipk3 in aortas when circHipk3 targeted to macrophages was overexpressed. **p* < 0.05 vs control group; n=3 per group (one-way ANOVA). (C) Macroscopic images of aortic characteristics of C57BL/6J mice with Ang II and circHipk3 targeted to macrophages overexpress constructs. (D-E) Representative immunostaining and densitometric analysis of suprarenal aortic NLRP3 protein expression in Ang II-induced AAA group or circHipk3 targeted to macrophages overexpress group (bars: upper 200 μm, lower 50 μm). **p*<0.05, n=5 per group (parametric unpaired t test). (bars: upper 200 μm, lower 50 μm).


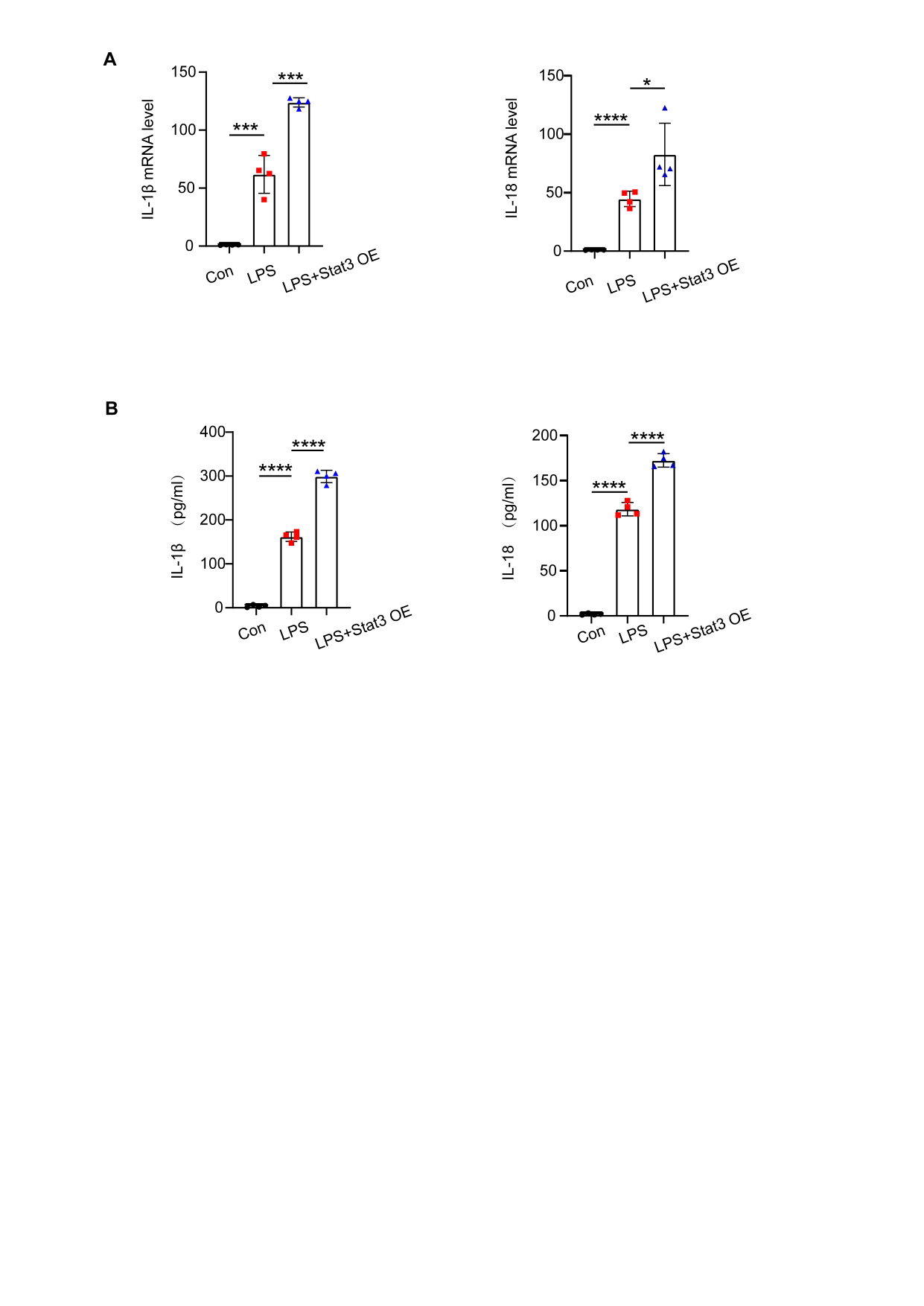
Supplemental Figure 11 . Overexpression of Stat3 promotes the transcription and secretion of IL-1β and IL-18 in macrophages.

BMDMs were transfected with a Stat3-overexpressing plasmid for a period of 24 hours, after which they were treated with lipopolysaccharide (LPS) at a concentration of 500 ng/ml for an additional 24 hours.

（A）Detect the mRNA levels of IL-1β and IL-18 in each group of cells by Q-PCR. **p*<0.05, *** *p*<0.001, **** *p*<0.0001,n=5 per group（B）Supernatants were collected for measuring lL-1β and lL-18 release by ELISA. **** *p*<0.0001,n=5 per group.


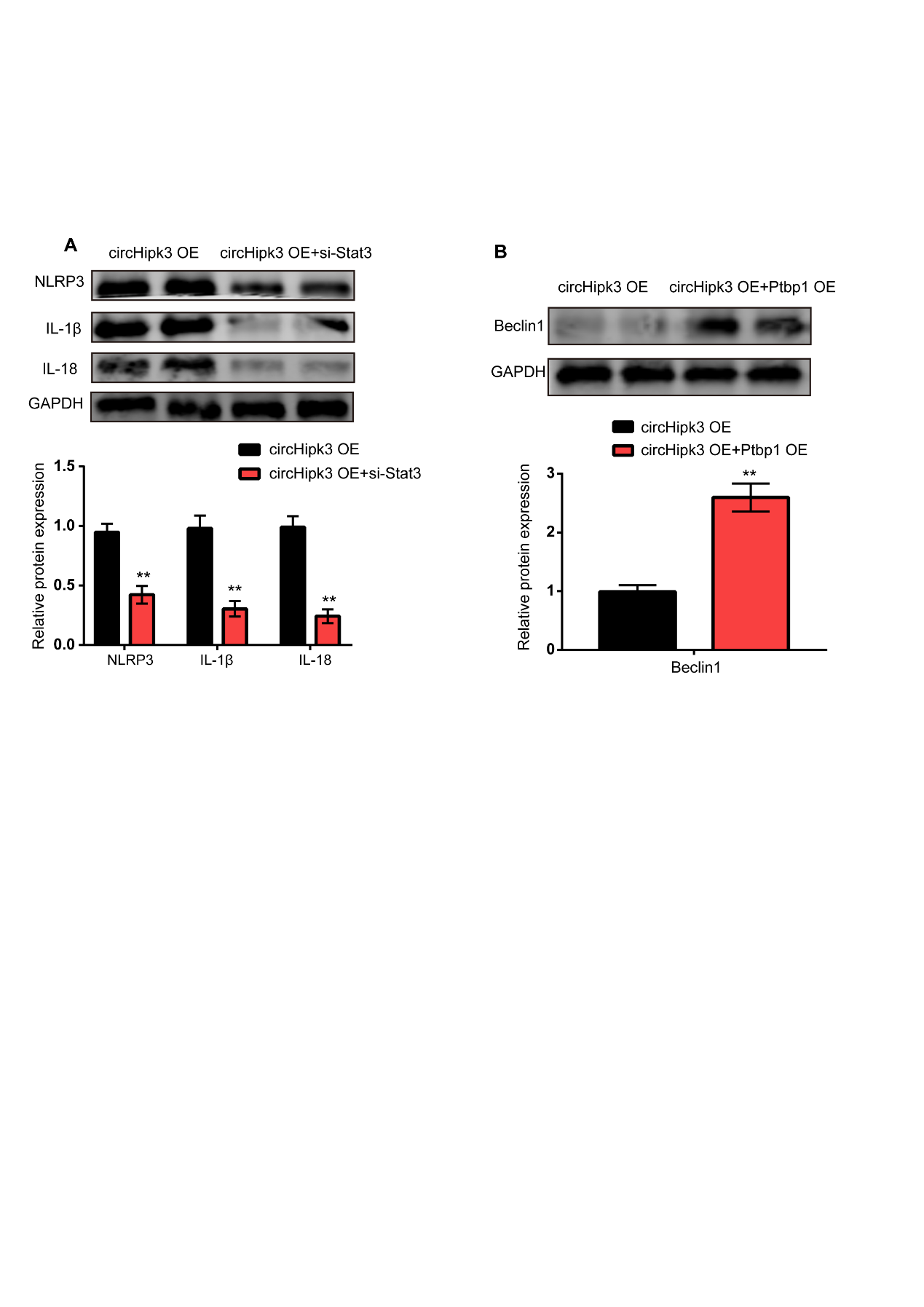
Supplemental Figure 12. Knockdown of Stat3 downregulates the levels of NLRP3 after overexpression of circHipk3, and promotion of Ptbp1 enhances the levels of Beclin1 after overexpression of circHipk3.

(A) Western blot analysis of the protein levels of NLRP3, IL-18, IL-1β in circHipk3 overexpression group or circHipk3 overexpression+stat3 knockdown group. ** *p*<0.01,n=5 per group.(GAPDH as the internal reference) . (B) Western blot analysis of the protein levels of beclin1 in circHipk3 overexpression group or circHipk3 overexpression+Ptbp1 overexpression group. ** *p*<0.01,n=5 per group. (GAPDH as the internal reference) .
